# Patient motivation as a predictor of digital health intervention effects: A meta-epidemiological study of cancer trials

**DOI:** 10.1371/journal.pone.0306772

**Published:** 2024-07-08

**Authors:** Yuqian Yan, Jesús López-Alcalde, Elena Stallings, Elena Jimenez Tejero, Claudia M. Witt, Jürgen Barth

**Affiliations:** 1 Institute for Complementary and Integrative Medicine, University Hospital Zurich and University of Zurich, Zurich, Switzerland; 2 Faculty of Medicine, Universidad Francisco de Vitoria (UFV), Madrid, Spain; 3 Unidad de Bioestadística Clínica, Instituto Ramón y Cajal de Investigación Sanitaria (IRYCIS), CIBERESP, Hospital Universitario Ramón y Cajal, Madrid, Spain; 4 Unidad de Bioestadística Clínica, Instituto Ramón y Cajal de Investigación Sanitaria (IRYCIS), Hospital Universitario Ramón y Cajal, Madrid, Spain; 5 Charité –Universitätsmedizin Berlin, Corporate Member of Freie Universität Berlin, Humboldt-Universität zu Berlin, and Berlin Institute of Health, Institute of Social Medicine, Epidemiology and Health Economics, Berlin, Germany; Debre Berhan University, ETHIOPIA

## Abstract

The objective of this meta-epidemiological study was to develop a rating that captures participants’ motivation at the study level in digital health intervention (DHI) randomised controlled trials (RCTs). The rating was used to investigate whether participants’ motivation is associated with the effect estimates in DHI RCTs for cancer patients. The development of the rating was based on a bottom-up approach involving the collection of information that captures participants’ baseline motivation in empirical studies from the Smartphone-RCCT Database. We specified three indicators for rating: indicator 1 captures whether the study team actively selects or enhances the motivation of the potential study participants; indicator 2 captures the study participants’ active engagement before the treatment allocation; and indicator 3 captures the potential bond and trust between the study participants and the person/institution referring to the study. The rating of each indicator and the overall rating varies between high motivation, moderate motivation, and low motivation. We applied the rating across 27 DHI RCTs with cancer patients. We performed meta-regression analysis to examine the effect of patient motivation on quality of life (QoL), psychological outcomes, and attrition. The intraclass correlation coefficient (ICC) indicated moderate to poor inter-rater reliability. The meta-regression showed that cancer patients’ overall motivation before engaging in the intervention was associated with the treatment effect of QoL. Patient motivation was not found to be associated with psychological outcomes or attrition. Subgroup analyses revealed that the clinical effects of DHIs were more prevalent in the high-motivation subgroups, whereas the low-motivation subgroups were unlikely to show intervention benefits. The likelihood of dropouts from DHIs seems to be especially high among the low-bond (indicator 3) subgroup. We suggest using single indicators since they reflect specific content. Better reporting about baseline motivation is required to enable meaningful interpretations in not only primary studies but also in evidence syntheses.

## Introduction

Patient motivation plays an important role in disease management, particularly for patients with chronic health conditions who have to actively engage in and maintain health-enhancing behaviours (i.e., self-management) [1]. For chronic diseases, such as cancer, patients with higher motivation have better treatment adherence, improved treatment outcomes, and lower attrition rates [2–4]. Therefore, patient motivation has been considered a key factor for successful therapeutic interventions [5].

Digital health interventions (DHIs) show vast potential in promoting health behaviours and supporting patients and health care systems [6–9], as they enable self-management among patients with chronic conditions. However, many DHIs experience a high level of attrition [10]. For example, a previous large-scale DHI study had substantial dropout rates, with a mean engagement of only 4.1 days [11]. A systematic review of randomised controlled trials (RCTs) examining DHIs reported a pooled attrition rate of 24.1% at short-term follow-up and 35.5% at longer-term follow-up [12]. One of the reasons why participants do not engage with DHIs may be a lack of motivation at the start of the trial [13]. Moreover, it can be assumed that motivated participants are more likely to adhere to and engage with the DHIs consistently, therefore enhancing the effectiveness of the intervention. In this regard, it is necessary to take note of patient motivation in self-care DHI trials. However, participants’ baseline motivation is rarely examined in DHI trials since there are few tools for measuring motivation in clinical settings [14]. It is not well understood how baseline motivation, as an effect modifier, influences the effectiveness and attrition rates in DHIs trials. To address this gap, study-level information can be utilized as proxies to develop a rating for participants’ motivation, enabling researchers to draw inferences about baseline motivation and the respective consequences on treatment outcomes.

The concept of patients’ motivation is ambiguous in most measurement tools; furthermore, the definition of motivation varies across different types of diseases and is influenced by different social and cultural backgrounds [14–17]. In our study, the definition of participants’ motivations closely aligns with Deci and Ryan’s self-determination theory on “intrinsic motivation” [18, 19], which are internal drives such as core values and interests that influence participants’ own behaviour [20, 21]. We define participants’ motivation as their self-determination and intrinsic drive to carry out tasks of the offered DHI in order to achieve therapeutic goals. We are especially interested in an individual’s intrinsic motivation before participating in an intervention of a specific trial. We specifically look for proxies for participants’ intrinsic motivation in the recruitment and screening process (e.g., interpersonal bond and trust, committed effort), and we assess participants’ characteristics (e.g., expectations, inherent interest, personal values and goals, self-efficacy). We do not consider the motivation that is associated with extrinsic rewards (e.g., financial benefits) or individuals’ conflicts (e.g., social pressure, shame).

The first objective of this meta-epidemiological study was to develop a rating that captures participants’ motivation at the study level in DHI RCTs. The second objective was to use this rating across a sample of studies to investigate whether participants’ motivation is associated with the effect estimates in DHI RCTs for cancer patients.

## Methods

The study protocol [22] was prospectively registered on the Open Science Framework and is available at https://osf.io/8ns2q. The registration also contains the appendices detailing the rating manual and the rating tree diagram.

### The purpose of the rating

The rating was developed to capture motivation of a study sample enrolled in a DHI RCT (regardless of the control condition). The DHI should involve self-care, where participants play an active role in disease management. This means that DHIs relying solely on passive tracking devices were not eligible. Despite the DHI is a self-care intervention, human guidance was allowed if this was facilitated indirectly through electronic channels.

### Development of the rating

We extracted information about recruitment and baseline characteristics from a sample of 20 primary studies in in the Smartphone-RCCT Database [23] (https://osf.io/nxerf/), which is hosted by our Institute. These 20 studies were selected based on different recruitment strategies to reflect the variability during the enrolment of patients. The development of the rating of participants’ motivation is based on a bottom-up approach by collecting a variety of study-level information. We identified different aspects of recruitment strategies and baseline characteristics from these 20 studies that might be associated with participants’ intrinsic motivation. Since the reporting of some potentially useful aspects for rating was weak, we ultimately specified three indicators serving as proxies that capture participants’ motivation at baseline and are likely to be rated across all studies.

The first indicator (labelled “expectation”) captures whether the study team actively selects or enhances the motivation of the potential study participants. We assumed that the selection of highly motivated participants or the active communication to increase participants’ motivation or expectations is associated with better treatment outcomes [24].

The second indicator (labelled “effort”) captures study participants’ active engagement before the treatment allocation. We assumed that a higher demand for participants’ investment (e.g., time, effort) before allocation reflects higher motivation among the participants, which is associated with better treatment outcomes [25].

The third indicator (labelled “bond”) captures the potential bond and trust between study participants and the person/institution referring to the study. We assumed that a stronger bond and higher level of trust reflects higher motivation among the participants, which is associated with better treatment outcomes [26].

The detailed description of these three indicators and the respective rating guidance are provided in the rating manual (S1 Appendix). The rating of each indicator varies between high motivation, moderate motivation, and low motivation. The first and second indicators have the same weight for the overall rating, and the third indicator has lower weight for the overall rating. The rationale for the lower weight of the third indicator was that the assessment of the bond (indicator 3) might be based on more indirect information, whereas the rating of both other indicators might rely on more direct information. Based on these premises, JB and YY developed a rating decision tree (S1 Fig) that considers all the rating patterns from the three indicators to generate an overall rating of participants’ motivation at the study level. The overall motivation can range from high to moderate to low.

### Study sample for rating

We applied the rating to RCTs that were included in a recently published systematic review and meta-analysis about the effect of mHealth app interventions on quality of life (QoL) and psychological outcomes in cancer patients [27]. The control groups in all studies received usual care, including waitlist control, conventional care, or health education delivered without the use of the mHealth app. We excluded three studies from our study for the following reasons: in one study, the target person for the app was the caregiver and not the patient [28]; one study used a dismantling study design [29] and did not meet our inclusion criteria [30]; and another study used only an educational intervention and did not require an active engagement of the patients [31].

### Rating and data extraction

Before applying the rating, JB and YY conducted training sessions with EJ and ES (training record in S2 Appendix) based on a training manual. Afterwards, a pilot testing with five RCTs from the study sample was undertaken to ensure consistency between these four raters regarding the judgement of the available study-level information. Once the training was completed and agreement had been achieved, all eligible studies were then rated by these four raters independently, and the results were entered into a predesigned rating form. The raters recorded their certainty of the rating for each indicator independently and made notes about the reasoning behind their rating and their certainty. The certainty of the rating has three levels: high certainty, moderate certainty, and low certainty. Disagreements were discussed between four raters (EJ, ES, JB, YY) and resolved by a final consensus between two raters (JB, YY). The consensus rating was not a standardized overruling procedure since all concerns of each rater were considered as valid. The consensus can be considered as re-rating based on the most comprehensive information taking the confidence of the rating by each raters into consideration. The final consensus of the rating was used as the predictor for meta-regression.

One author (YY) extracted information about treatment outcomes from the tables, flow diagram, and text of the included studies. The following outcomes were extracted: QoL, anxiety, depression, and attrition rate for each study arm. All measurement tools for QoL, anxiety, and depression were allowed. When multiple measurement tools were used for one outcome in the same study, we considered the data that had the lowest attrition. The attrition rate was defined as the proportion of individuals who dropped out during the intervention, which corresponds to those who disengaged from or ceased their involvement in the DHI itself (intervention dropouts). This definition as reported in the study flow chart, does not mean dropouts due to death or individuals who merely discontinued questionnaire assessments. For all outcomes, we extracted data from the first post-intervention time point. If the intervention period was unclear, we considered the measurement taken at the longest follow-up reported in the included study. A second author (JB) crosschecked the extracted data.

### Statistical analysis

In order to get an impression about the performance of the rating, we measured the inter-rater reliability of four raters by calculating the intraclass correlation coefficient (ICC) [32, 33]. Additionally, we calculated the ICC for five raters, treating the consensus rating as the fifth rater. The ICC value ranges between 0 and 1. Values less than 0.5 indicate poor agreement; values between 0.5 and 0.75 indicate moderate agreement; values between 0.75 and 0.9 indicate good agreement; and values equal to or greater than 0.90 indicate excellent agreement [32]. We also explored the correlations between the three indicators and the correlations between each indicator and the overall motivation [34].

As outcome measure in the meta-analysis of health outcomes, we used the standardized mean difference (SMD) [35] with a 95% confidence interval (CI) to estimate intervention effects on QoL, depression, and anxiety. If SDs were not provided, they were calculated using the available data [36]. If there were no available data to obtain SDs, the baseline SDs were used as post-intervention SDs for the meta-analysis. The risk ratio (RR) with a 95% CI was used as effect estimates for attrition.

For the meta-analytic procedure we used the random effects model with Knapp–Hartung adjustment for pooling of studies [37, 38]. We then fitted a mixed effects meta-regression model to the rating variables (i.e., overall motivation and single indicators) to examine the association between different levels of participants’ motivation and the treatment outcomes. A two-sided *P* < .05 was used to indicate a statistically significant difference in the overall effect. We assessed statistical heterogeneity with Cochran’s Q test and measured its magnitude with Higgin’s and Thompson’s *I*^*2*^ statistics, where *I*^*2*^ ≥ 50% indicated substantial heterogeneity [39]. All statistical analyses were conducted using R (R Foundation for Statistical Computing, Vienna, Austria, version 4.3.1) [40].

## Results

A total of 27 RCTs with 4,986 cancer patients were included in our study (S3 Appendix). The characteristics of these studies can be found in the original systematic review [27]. The ratings of the study participants’ motivations were as follows: for overall motivation, 19 studies (70%) were rated as high motivation, five studies (19%) were rated as moderate motivation, and three studies (11%) were rated as low motivation. For indicator 1 (expectation), 16 studies (59%) were rated as high motivation, 11 studies (41%) were rated as moderate motivation, and no study was rated as low motivation. For indicator 2, 12 studies (44%) were rated as high motivation, seven studies (26%) were rated as moderate motivation, and eight studies (30%) were rated as low motivation. For indicator 3, 13 studies (48%) were rated as high motivation, three studies (11%) were rated as moderate motivation, and 11 studies (41%) were rated as low motivation. The certainty levels from four raters, both on a per-study basis and in summary form, can be found in S4 and S5 Appendices.

### Inter-rater reliability

The ICC based on the rating of four raters are 0.49 for the overall rating, 0.55 for Indicator 1, 0.42 for Indicator 2, and 0.27 for Indicator 3. The ICC results based on five ratings, where the consensus rating is considered an independent rater, are provided in S6 Appendix. There results showed higher reliability compared to the initial rating with only four raters.

### Meta-regression and subgroup analysis

#### QoL

Twenty-two studies (81%) reported about QoL (Table 1 and S2 Fig). The meta-analysis showed that mHealth app interventions improved QoL among cancer patients compared to usual care (SMD = 0.31; 95% CI, 0.17 to 0.46; *P* < 0.001), with high heterogeneity between the studies (*I*^*2*^ = 64%; chi-square *P* < 0.001). Meta-regression showed that cancer patients’ overall motivation was associated with the treatment effect of QoL (*P* < 0.001), and the effect estimate was the largest in the moderate motivation subgroups. Subgroup analysis based on overall motivation suggested that mHealth app interventions improved QoL only in the high- and moderate-motivation subgroups. Subgroup analyses of pooled estimates by single indicators revealed that mHealth app interventions were consistently beneficial in improving QoL in the high-motivation subgroups, while the interventions were consistently inconclusive in the low-motivation subgroups. In addition, subgroup analyses showed low heterogeneity among studies with patients with high and moderate overall motivation (21% and 0%, respectively), high expectation (indicator 1) (34%), high and moderate effort (indicator 2) (18% and 30%, respectively), and moderate bond (indicator 3) (32%).

**Table 1:** Meta-regression and subgroup analysis on QoL.

Characteristics	Studies	SMD	95% CI		Heterogeneity		Meta-regression
			LL	UL	*I*^*2*^ (%)	*P*	*P*
**All studies**	22	0.31	0.17	0.46	64	< 0.001	
**Overall motivation**							< 0.001
High motivation	17	0.32	0.24	0.40	21	0.211	
Moderate motivation	3	0.80	0.31	1.30	0	0.437	
Low motivation	2	-0.33	-3.49	2.83	66	0.086	
**Indicator 1 (expectation)**							0.886
High motivation	13	0.32	0.22	0.41	34	0.112	
Moderate motivation	9	0.33	-0.03	0.70	80	< 0.001	
Low motivation	0						
**Indicator 2 (effort)**							0.872
High motivation	12	0.33	0.23	0.43	18	0.267	
Moderate motivation	5	0.28	0.07	0.49	30	0.223	
Low motivation	5	0.32	-0.49	1.14	90	< 0.001	
**Indicator 3 (bond)**							0.367
High motivation	10	0.42	0.21	0.62	61	0.007	
Moderate motivation	3	0.21	-0.29	0.71	32	0.232	
Low motivation	9	0.22	-0.06	0.51	70	< 0.001	

**Notes**: SMD greater than zero indicates an increase in QoLfavouring the mHealth app intervention group. The meta-regression *P* value indicates differences between the subgroups (high, moderate, low).

**Abbreviations**: SMD, standardized mean difference; CI, confidence interval; LL, lower limit; UL, upper limit.

#### Anxiety

Twelve studies (44%) reported about anxiety (Table 2 and S3 Fig). The meta-analysis showed that mHealth app interventions reduced anxiety among cancer patients compared to usual care (SMD = -0.82; 95% CI, -1.55 to -0.10; *P* = 0.030), with high heterogeneity between the studies (*I*^*2*^ = 95%; chi-square *P* < 0.001). No association was found between different levels of patients’ motivation and anxiety. Subgroup analyses based on overall motivation and expectation (indicator 1) showed that the mHealth app interventions decreased anxiety only in the high-motivation subgroups. Subgroup analyses based on single indicators revealed that the treatment effect of mHealth app interventions was consistently inconclusive in the low-motivation subgroups. In addition, the heterogeneity remained high in all subgroup analyses.

**Table 2:** Meta-regression and subgroup analysis on anxiety.

Characteristics	Studies	SMD	95% CI		Heterogeneity		Meta-regression
			LL	UL	*I*^*2*^ (%)	*P*	*P*
**All studies**	12	-0.82	-1.55	-0.10	95	< 0.001	
**Overall motivation**							0.200
High motivation	8	-0.71	-1.20	-0.23	89	< 0.001	
Moderate motivation	2	0.03	-4.96	5.02	85	0.01	
Low motivation	2	-2.07	-26.52	22.39	99	< 0.001	
**Indicator 1 (expectation)**							0.534
High motivation	8	-0.66	-1.23	-0.08	91	< 0.001	
Moderate motivation	4	-1.15	-4.15	1.85	98	< 0.001	
Low motivation	0						
**Indicator 2 (effort)**							0.563
High motivation	5	-0.46	-0.99	0.06	79	< 0.001	
Moderate motivation	3	-0.63	-1.53	0.28	65	0.059	
Low motivation	4	-1.38	-4.53	1.77	98	< 0.001	
**Indicator 3 (bond)**							0.891
High motivation	6	-0.65	-1.32	0.03	90	< 0.001	
Moderate motivation	1	-1.21	-1.80	-0.63	NA	NA	
Low motivation	5	-0.96	-3.11	1.20	98	< 0.001	

**Notes**: SMD less than zero indicates a reduction in anxiety favouring the mHealth app intervention group. The meta-regression *P* value indicates differences between subgroups (high, moderate, low).

**Abbreviations**: SMD, standardized mean difference; CI, confidence interval; LL, lower limit; UL, upper limit; NA, not applicable.

#### Depression

Eleven studies (41%) reported about depression (Table 3 and S4 Fig). For the pooled analysis, no significant difference was found between the mHealth app intervention and usual care (SMD = -0.60; 95% CI, -1.37 to 0.16; *P* = 0.110). In addition, significant heterogeneity existed between the studies (*I*^*2*^ = 94%; chi-square *P* < 0.001). No association was found between different levels of patients’ motivation and depression. Subgroup analyses based on overall motivation and expectation (indicator 1) showed that the mHealth app interventions decreased depression only in the high-motivation subgroups. Subgroup analyses based on single indicators revealed that the treatment effect of mHealth app interventions was consistently inconclusive in the low-motivation subgroups. The heterogeneity remained high in all subgroup analyses.

**Table 3:** Meta-regression and subgroup analysis on depression.

Characteristics	Studies	SMD	95% CI		Heterogeneity		Meta-regression
			LL	UL	*I*^*2*^ (%)	*P*	*P*
**All studies**	11	-0.60	-1.37	0.16	94	< 0.001	
**Overall motivation**							0.140
High motivation	7	-0.41	-0.68	-0.13	48	0.071	
Moderate motivation	2	0.18	0.14	0.22	0	0.982	
Low motivation	2	-2.01	-26.46	22.44	99	< 0.001	
**Indicator 1 (expectation)**							0.567
High motivation	6	-0.39	-0.76	-0.02	62	0.022	
Moderate motivation	5	-0.85	-3.01	1.31	97	< 0.001	
Low motivation	0						
**Indicator 2 (effort)**							0.549
High motivation	5	-0.43	-0.90	0.04	65	0.021	
Moderate motivation	3	-0.23	-1.04	0.58	60	0.081	
Low motivation	3	-1.27	-6.99	4.44	99	< 0.001	
**Indicator 3 (bond)**							0.814
High motivation	4	-0.31	-0.73	0.11	55	0.086	
Moderate motivation	1	-1.00	-1.56	-0.43	NA	NA	
Low motivation	6	-0.76	-2.41	0.89	97	< 0.001	

**Notes**: SMD less than zero indicates a reduction in depression favouring the mHealth app intervention group. The meta-regression *P* value indicates differences between the subgroups (high, moderate, low).

**Abbreviations**: SMD, standardized mean difference; CI, confidence interval; LL, lower limit; UL, upper limit; NA, not applicable.

#### Attrition

Twenty-three studies (85%) reported about attrition (Table 4 and S5 Fig). The meta-analysis showed that patients in the mHealth app intervention group had a higher likelihood of dropping out from the study as compared to the control group (RR = 1.66; 95% CI, 1.01 to 2.71; *P* = 0.045), with significant heterogeneity between the studies (*I*^*2*^ = 73%, chi-square *P* < 0.001). No association was found between different levels of patient motivation and attrition. Subgroup analyses showed that the risk of attrition was the highest for the mHealth interventions in the low-motivation subgroup when grouped by bond (indicator 3) (RR = 2.87; 95% CI, 1.37 to 6.00). In addition, subgroup analyses showed low heterogeneity for moderate and low overall motivation (35% and 0%, respectively), low effort (indicator 2) (15%), and moderate bond (indicator 3) (0%).

**Table 4:** Meta-regression and subgroup analysis on attrition.

Characteristics	Studies	RR	95% CI		Heterogeneity		Meta-regression
			LL	UL	*I*^*2*^ (%)	*P*	*P*
**All studies**	23	1.66	1.01	2.71	73	< 0.001	
**Overall motivation**							0.794
High motivation	17	1.64	0.88	3.08	78	< 0.001	
Moderate motivation	3	2.98	0.05	188.78	35	0.217	
Low motivation	3	1.22	0.27	5.62	0	0.392	
**Indicator 1 (expectation)**							0.401
High motivation	14	1.98	0.96	4.06	80	< 0.001	
Moderate motivation	9	1.27	0.59	2.72	42	0.087	
Low motivation	0						
**Indicator 2 (effort)**							0.905
High motivation	12	1.81	0.86	3.81	64	< 0.001	
Moderate motivation	7	1.35	0.38	4.83	80	< 0.001	
Low motivation	4	1.45	0.41	5.10	15	0.317	
**Indicator 3 (bond)**							0.115
High motivation	9	1.09	0.42	2.81	65	0.003	
Moderate motivation	3	0.96	0.70	1.33	0	0.81	
Low motivation	11	2.87	1.37	6.00	57	0.01	

**Notes**: RR greater than one indicates an increase in the risk of dropout among the mHealth intervention group compared to the usual care group. The meta-regression *P* value indicates differences between the subgroups (high, moderate, low).

**Abbreviations**: RR, risk ratio; CI, confidence interval; LL, lower limit; UL, upper limit.

## Discussion

In this meta-epidemiological study, we successfully developed a rating for participants’ motivation in DHI trials. We applied the rating to a sample of mHealth app intervention studies, rated by four raters, showing moderate to poor reliability. However, the reliability with the final consensus and the initial decision of the four raters showed better agreement. Our findings demonstrated an association between cancer patients’ overall motivation before engaging in the intervention and treatment effects for QoL. We did not find an association between patients’ motivation and psychological outcomes or attrition. However, our subgroup analyses revealed that mHealth app interventions were unlikely to show clinical benefits in the low-motivation subgroups in general. The likelihood of attrition from mHealth app interventions seems to be high in the low-bond (indicator 3) subgroup.

In our study sample, patients’ overall motivation was found to be associated with the treatment effect of QoL. However, it is important to interpret this finding cautiously. The overall motivation was determined based on an algorithm of different rating patterns of three indicators, as outlined in our prospectively registered protocol [22]. Specifically, indicator 3 has lower weight as compared to indicator 1 and 2, considering that the assessment of Indicator 3 might rely more on indirect study-level information. Using this algorithm, we classified 19 studies (70%) as having high motivation, five studies (19%) as having moderate motivation, and three studies (11%) as having low motivation. While we maintain confidence in the rationale behind our initial decision, we also acknowledge its arbitrary nature. As suggested by one reviewer, we re-calculated the overall motivation by assigning equal weight to all three indicators (scale of 1 to 3, 1 for low motivation and 3 for high motivation). We summed the scores of the three indicators (range is 3 to 9) and established meaningful cut-off scores. We used score 3, 4 or 5 for low motivation, score 6 or 7 as moderate motivation, and 8 or 9 as high motivation. This new approach resulted in 11 studies (41%) classified as high motivation, 11 studies (41%) as moderate motivation, and five studies (19%) as low motivation. Subsequently, we conducted a sensitivity analysis for all investigated outcomes. As expected, the results of meta-regression changed significantly for QoL and attrition, as these two outcomes included most studies and the distribution of studies across subgroups changes in the sensitivity analysis. The result of the sensitivity analysis is available in S7 Appendix. Nevertheless, both the primary analysis and the sensitivity analysis suggested a similar pattern that low motivated patients did not benefit from DHI self-care intervention.

Combining indicators into an overall score has the advantage to allow a single regression analysis without multiple testing [41] and may avoid contradicting results per indicator. Therefore, we used both the overall score and single indicators in the regression models. Using the overall score might be intuitive since motivational aspects may add up, but as said before the weighting of indicators may be considered arbitrary. Using single indicators might be justified since the indicators had quite low correlations (0.03 < r < 0.28) with each other and may capture different aspects of motivation, but the number of tests considerably increases. From a conceptual point of view, a closer look at the advantages and disadvantages of scoring in meta-analyses must be taken: Peter Juni et al. [42] showed in a meta-epidemiological study that the association of “study quality” and treatment benefits depends on the scale used to assess study quality. It can be either positive or negative, which makes the use of scores questionable. Therefore, we suggest using single indicators since they reflect specific content (i.e., expectations, effort, bond), and the weighting of indicators can be avoided.

When looking at the single indicators, we found that in studies with patients having a low bond to the treatment referrer, the percentage of dropouts during the study was higher, which is in line with earlier research [43]. This supports the idea that trustworthy physicians or health care professionals can serve as powerful advocates for successful clinical research. According to a survey [44], 84% of patients implied that they would consider participating in a clinical trial if their physician recommended it.

According to CONSORT-EHEALTH [45], it is highly recommended to specify how participants were briefed during recruitment and in informed consent procedures. This information can influence user self-selection, expectations, and may introduce bias into the results (refer to Checklist 4a: Eligibility criteria for participants). However, our rating refers to available indirect study-level information about participants’ expectations, effort, and bonds to reflect motivational baseline characteristics in DHI trials. Based on our experience in this study, such an indirect approach is necessary since the motivation of participants who are enrolled in DHI trials were rarely reported. Since such aspects could be assessed, DHI trialists should be encouraged to report information about patients’ expectations, beliefs, previous treatment experiences, and preferences. This may also be useful for understanding heterogeneity between trials’ findings, since study authors mention low motivation as a reason for the low effectiveness of some DHIs. The most commonly implemented measures are scales about digital health literacy (DHL) or affinity to technology [46], which reflects competence and knowledge but does not directly relate to motivation. Some populations have low DHL [47], but increasing DHL is possible [48]. Higher DHL may increase the benefits of DHIs, as shown for health literacy [49]. Similar to research on DHL, patients’ motivation should be assessed before the uptake of a DHI in the trial.

### Clinical implications

Health care providers have an important role in decision-making to support patients in their choice of the most suitable and promising DHI. If health care providers are aware of low motivation in patients, interventions to increase patients’ motivation and expectations by addressing patients’ concerns would be an option [50]. By engaging patients in such discussions (e.g., support, education, encouragement), clinicians may increase patients’ motivation and treatment benefits.

### Research implications

The implementation of measures to assess patients’ motivation in DHI trials would allow the stratification for motivation in RCTs to prevent imbalance between groups. Baseline information about motivation could also be integrated as an interaction factor for effectiveness analyses and, consequently, could also be used as a moderator to explain heterogeneity between trials in systematic reviews.

Furthermore, we would also encourage a better assessment and a more transparent reporting in DHI trials regarding specific indicators (i.e., expectations, effort, bond). Regarding expectations, specific measures are available [51, 52]. Regarding effort, a clearer reporting on participants’ active engagement before the start of the trial is desirable. Concerning bond, information about the recruitment strategy including how and by whom participants are being approached would be of help in order to get information about working alliance [53].

Out study developed and applied a rather complex rating. In order to achieve a common understanding of the concepts, it is required to have thorough training sessions in advance and also extensive consensus meetings. Both steps are necessary to foster a common understanding and to mitigate any inherent biases among raters, thereby making the final rating meaningful.

### Strengths and limitations

Four raters were involved in the rating of all studies. Despite the fact, that some studies did not report much about motivational issues of the study sample, we achieved good procedures to deal with a lack of information with moderate to poor reliable ratings. Nevertheless, some limitations should be taken into consideration. First, studies in our sample were not evenly distributed over subgroups; as a matter of fact, the majority of studies have overall high-motivation patients (70%). Second, weighting of indicators in a composite score of overall motivation may be arbitrary. Third, some studies had large effect sizes and could be considered as outliers in the meta-regression. Forth, high heterogeneity remained in some subgroups. Finally, the rating decision tree might not reflect the true hierarchy between the three indicators.

## Conclusion

The clinical effects of DHI were more prevalent in the high-motivation subgroups, whereas the treatment was inclusive in the low-motivation subgroups. The likelihood of dropouts from DHI seems to be especially high in the low-bond (indicator 3) subgroup. We suggest using single indicators since they reflect specific content. Better reporting about baseline motivation is required to allow for meaningful interpretation in not only primary studies but also in evidence synthesis.

## Supporting information

S1 Appendix Rating manual for patient motivation. (DOCX)

S2 Appendix Training record of the raters. (DOCX)

S3 Appendix Included studies for rating. (DOCX)

S4 Appendix Certainty levels of 4 raters per studies. (DOCX)

S5 Appendix Summary of certainty levels per raters. (DOCX)

S6 Appendix ICC based on five ratings (four raters and consensus) for the overall rating and each indicator. (DOCX)

S7 Appendix Sensitivity analysis: Equal weighting of indicators for overall motivation. (DOCX)

S1 Fig Rating decision tree. The rating tree is used to generate an overall rating of patient motivation at the study level based on the rating patterns from the three indicators.
(PDF)

S2 Fig Forest plot. Meta-analysis on quality of life.
(PNG)

S3 Fig Forest plot. Meta-analysis on anxiety.
(PNG)

S4 Fig Forest plot. Meta-analysis on depression.
(PNG)

S5 Fig Forest plot. Meta-analysis on attrition.
(PNG)

S1 Checklist (DOCX)

## References

[1] Lorig KR, Holman H. Self-management education: history, definition, outcomes, and mechanisms. Ann Behav Med. 2003;26(1):1–7. doi: 10.1207/S15324796ABM2601_01 12867348

[2] Carl Simonton O, Matthews-Simonton S, Flint Sparks T. Psychological intervention in the treatment of cancer. Psychosomatics. 1980;21(3):226–33. doi: 10.1016/s0033-3182(80)73697-6 7367569

[3] Cox LS, Patten CA, Ebbert JO, Drews AA, Croghan GA, Clark MM, et al. Tobacco use outcomes among patients with lung cancer treated for nicotine dependence. Journal of Clinical Oncology. 2002;20(16):3461–9. doi: 10.1200/JCO.2002.10.085 12177107

[4] Gönderen Çakmak HS, Kapucu S. The effect of educational follow-up with the motivational interview technique on self-efficacy and drug adherence in cancer patients using oral chemotherapy treatment: A randomized controlled trial. Seminars in Oncology Nursing. 2021;37(2):151140. doi: 10.1016/j.soncn.2021.151140 33766423

[5] Ryan RM, Deci EL. Self-determination theory: basic psychological needs in motivation, development, and wellness. New York, NY, US: The Guilford Press; 2017. xii, 756–xii, p.

[6] Blok AC, Sadasivam RS, Amante DJ, Kamberi A, Flahive J, Morley J, et al. Gamification to motivate the unmotivated smoker: the “take a break” digital health intervention. Games Health J. 2019;8(4):275–84. doi: 10.1089/g4h.2018.0076 31219347 PMC6686688

[7] Liu F, Kong X, Cao J, Chen S, Li C, Huang J, et al. Mobile phone intervention and weight loss among overweight and obese adults: a meta-analysis of randomized controlled trials. Am J Epidemiol. 2015;181(5):337–48. doi: 10.1093/aje/kwu260 25673817 PMC4339384

[8] Widmer RJ, Collins NM, Collins CS, West CP, Lerman LO, Lerman A. Digital health interventions for the prevention of cardiovascular disease: a systematic review and meta-analysis. Mayo Clinic Proceedings. 2015;90(4):469–80. doi: 10.1016/j.mayocp.2014.12.026 25841251 PMC4551455

[9] Jones KR, Lekhak N, Kaewluang N. Using mobile phones and short message service to deliver self-management interventions for chronic conditions: a meta-review. Worldviews Evid Based Nurs. 2014;11(2):81–8. doi: 10.1111/wvn.12030 24597522

[10] Eysenbach G. The law of attrition. J Med Internet Res. 2005;7(1):e11. doi: 10.2196/jmir.7.1.e11 15829473 PMC1550631

[11] Hershman SG, Bot BM, Shcherbina A, Doerr M, Moayedi Y, Pavlovic A, et al. Physical activity, sleep and cardiovascular health data for 50,000 individuals from the MyHeart Counts Study. Scientific Data. 2019;6(1):24. doi: 10.1038/s41597-019-0016-7 30975992 PMC6472350

[12] Linardon J, Fuller-Tyszkiewicz M. Attrition and adherence in smartphone-delivered interventions for mental health problems: a systematic and meta-analytic review. J Consult Clin Psychol. 2020;88(1):1–13. doi: 10.1037/ccp0000459 31697093

[13] Schroé H, Crombez G, De Bourdeaudhuij I, Van Dyck D. Investigating when, which, and why users stop using a digital health intervention to promote an active lifestyle: secondary analysis with a focus on health action process approach-based psychological determinants. JMIR Mhealth Uhealth. 2022;10(1):e30583. doi: 10.2196/30583 35099400 PMC8845016

[14] Hosseini F, Alavi NM, Mohammadi E, Sadat Z. Scoping review on the concept of patient motivation and practical tools to assess it. Iran J Nurs Midwifery Res. 2021;26(1):1–10. doi: 10.4103/ijnmr.IJNMR_15_20 33954092 PMC8074736

[15] Roberts JR, Maxfield M. Examining the relationship between religious and spiritual motivation and worry about alzheimer’s disease in later life. J Relig Health. 2018;57(6):2500–14. doi: 10.1007/s10943-018-0635-x 29730806

[16] Zhang R, Eschler J, Reddy M. Online support groups for depression in China: culturally shaped interactions and motivations. Computer Supported Cooperative Work (CSCW). 2018;27(3):327–54.

[17] Pargament KI. Spirituality as an irreducible human motivation and process. International Journal for the Psychology of Religion. 2013;23(4):271–81.

[18] Deci EL, Ryan RM. Intrinsic motivation and self-determination in human behavior. New York: Plenum Publishing Co; 1985.

[19] Deci EL, Ryan RM. The general causality orientations scale: self-determination in personality. Journal of Research in Personality. 1985;19(2):109–34.

[20] Flannery M. Self-determination theory: intrinsic motivation and behavioral change. Oncol Nurs Forum. 2017;44(2):155–6. doi: 10.1188/17.ONF.155-156 28222078

[21] Ackerman C. Self determination theory and how it explains motivation (updated Feburary 2024). Available from: https://positivepsychology.com/self-determination-theory/.

[22] Yan Y, Lopez-Alcalde J, Witt C, Barth J. Participants’ motivation as predictor of treatment outcomes in mHealth studies: a meta-epidemiological study protocol. doi: 10.17605/OSF.IO/8NS2Q2023

[23] Barth J, Wang J, Lopez-Alcalde J, Kramm C, Pach D, Álvarez-Díaz N, et al. Smartphone-RCCT: an online repository of randomized controlled clinical trials of smartphone applications for chronic conditions. Trials. 2022;23(1):909. doi: 10.1186/s13063-022-06849-x 36303168 PMC9615349

[24] Laferton JAC, Oeltjen L, Neubauer K, Ebert DD, Munder T. The effects of patients’ expectations on surgery outcome in total hip and knee arthroplasty: a prognostic factor meta-analysis. Health Psychology Review. 2022;16(1):50–66. doi: 10.1080/17437199.2020.1854051 33228474

[25] Salamone JD, Yohn SE, López-Cruz L, San Miguel N, Correa M. Activational and effort-related aspects of motivation: neural mechanisms and implications for psychopathology. Brain. 2016;139(Pt 5):1325–47. doi: 10.1093/brain/aww050 27189581 PMC5839596

[26] Shaw BR, Han JY, Hawkins RP, Stewart J, McTavish F, Gustafson DH. Doctor–patient relationship as motivation and outcome: examining uses of an interactive cancer communication system. International Journal of Medical Informatics. 2007;76(4):274–82. doi: 10.1016/j.ijmedinf.2005.12.002 16460995

[27] Qin M, Chen B, Sun S, Liu X. Effect of mobile phone app-based interventions on quality of life and psychological symptoms among adult cancer survivors: systematic review and meta-analysis of randomized controlled trials. J Med Internet Res. 2022;24(12):e39799. doi: 10.2196/39799 36534460 PMC9808609

[28] Peng Z, Li L, Chen Y, Feng Z, Fang X. WeChat app-based reinforced education improves the quality of opioid titration treatment of cancer-related pain in outpatients: a randomized control study. BMC Cancer. 2020;20(1):852. doi: 10.1186/s12885-020-07270-w 32887560 PMC7472406

[29] Papa A, Follette WC. Dismantling studies of psychotherapy. The Encyclopedia of Clinical Psychology. p. 1–6.

[30] Lei L. The application of APP in the transitional care among the post-operative patients with laryngeal cancer. Dissertation of nursing school in Zhengzhou University: Zhengzhou University; 2016.

[31] Foley NM, O’Connell EP, Lehane EA, Livingstone V, Maher B, Kaimkhani S, et al. PATI: patient accessed tailored information: a pilot study to evaluate the effect on preoperative breast cancer patients of information delivered via a mobile application. Breast. 2016;30:54–8. doi: 10.1016/j.breast.2016.08.012 27611236

[32] Koo TK, Li MY. A guideline of selecting and reporting intraclass correlation coefficients for reliability research. J Chiropr Med. 2016;15(2):155–63. doi: 10.1016/j.jcm.2016.02.012 27330520 PMC4913118

[33] Shrout PE, Fleiss JL. Intraclass correlations: uses in assessing rater reliability. Psychol Bull. 1979;86(2):420–8. doi: 10.1037//0033-2909.86.2.420 18839484

[34] Turney S. Pearson correlation coefficient (r) | guide & examples. Scribbr. Retrieved August 30, 2023, from https://www.scribbr.com/statistics/pearson-correlation-coefficient/.

[35] Hedges LV. Distribution theory for glass’s estimator of effect size and related estimators. Journal of Educational and Behavioral Statistics. 1981;6(2):107–28.

[36] Higgins J, Li T, Deeks Je. Chapter 6: Choosing effect measures and computing estimates of effect. In: Higgins JPT, Thomas J, Chandler J, Cumpston M, Li T, Page MJ, Welch VA (editors). Cochrane Handbook for Systematic Reviews of Interventions version 6.3 (updated February 2022). Available from www.training.cochrane.org/handbook.2022.

[37] Langan D, Higgins JPT, Jackson D, Bowden J, Veroniki AA, Kontopantelis E, et al. A comparison of heterogeneity variance estimators in simulated random-effects meta-analyses. Res Synth Methods. 2019;10(1):83–98. doi: 10.1002/jrsm.1316 30067315

[38] Knapp G, Hartung J. Improved tests for a random effects meta-regression with a single covariate. Stat Med. 2003;22(17):2693–710. doi: 10.1002/sim.1482 12939780

[39] Deeks J, Higgins J, Altman De. Chapter 10: Analysing data and undertaking meta-analyses. In: Higgins JPT, Thomas J, Chandler J, Cumpston M, Li T, Page MJ, Welch VA (editors). Cochrane Handbook for Systematic Reviews of Interventions version 6.3 (updated February 2022). Available from www.training.cochrane.org/handbook.2022.

[40] R Core Team. R: A language and environment for statistical computing. R Foundation for Statistical Computing, Vienna, Austria. Available from https://www.R-project.org/2021.

[41] Higgins JP, Thompson SG. Controlling the risk of spurious findings from meta-regression. Stat Med. 2004;23(11):1663–82. doi: 10.1002/sim.1752 15160401

[42] Jüni P, Witschi A, Bloch R, Egger M. The hazards of scoring the quality of clinical trials for meta-analysis. Jama. 1999;282(11):1054–60. doi: 10.1001/jama.282.11.1054 10493204

[43] Henkemans OAB, Rogers WA, Dumay AMC. Personal characteristics and the law of attrition in randomized controlled trials of eHealth services for self-care. Gerontechnology. 2011;10(3):157–68.

[44] Getz K. Enabling healthcare providers as facilitators of patient engagement. Applied Clinical Trials. 2017;26(10).

[45] Eysenbach G. CONSORT-EHEALTH: improving and standardizing evaluation reports of Web-based and mobile health interventions. J Med Internet Res. 2011;13(4):e126. doi: 10.2196/jmir.1923 22209829 PMC3278112

[46] Franke T, Attig C, Wessel D. A Personal resource for technology interaction: development and validation of the affinity for technology interaction (ATI) scale. International Journal of Human–Computer Interaction. 2019;35(6):456–67.

[47] Estrela M, Semedo G, Roque F, Ferreira PL, Herdeiro MT. Sociodemographic determinants of digital health literacy: a systematic review and meta-analysis. Int J Med Inform. 2023;177:105124. doi: 10.1016/j.ijmedinf.2023.105124 37329766

[48] Dong Q, Liu T, Liu R, Yang H, Liu C. Effectiveness of digital health literacy interventions in older adults: single-arm meta-analysis. J Med Internet Res. 2023;25:e48166. doi: 10.2196/48166 37379077 PMC10365623

[49] Berkman ND, Sheridan SL, Donahue KE, Halpern DJ, Crotty K. Low health literacy and health outcomes: an updated systematic review. Ann Intern Med. 2011;155(2):97–107. doi: 10.7326/0003-4819-155-2-201107190-00005 21768583

[50] Bennett G. Miller, W. R. and Rollnick, S. (1991) Motivational interviewing: preparing people to change addictive behavior. New York: Guilford Press, 1991. Pp. xvii + 348. £24.95 hardback, £11.50 paper. ISBN 0–89862–566–1. Journal of Community & Applied Social Psychology. 1992;2(4):299–300.

[51] Laferton JA, Kube T, Salzmann S, Auer CJ, Shedden-Mora MC. Patients’ expectations regarding medical treatment: a critical review of concepts and their assessment. Front Psychol. 2017;8:233. doi: 10.3389/fpsyg.2017.00233 28270786 PMC5318458

[52] Barth J, Kern A, Lüthi S, Witt CM. Assessment of patients’ expectations: development and validation of the expectation for treatment scale (ETS). BMJ Open. 2019;9(6):e026712. doi: 10.1136/bmjopen-2018-026712 31213446 PMC6585827

[53] Farin E, Gramm L, Schmidt E. The patient-physician relationship in patients with chronic low back pain as a predictor of outcomes after rehabilitation. J Behav Med. 2013;36(3):246–58. doi: 10.1007/s10865-012-9419-z 22476813

