# Supplementary material for: Patient motivation as a predictor of digital health intervention effects: A meta-epidemiological study of cancer trials
Source: PLoS One. 2024 Jul 8;19(7):e0306772. doi: 10.1371/journal.pone.0306772 (PMC11230537; doi:10.1371/journal.pone.0306772)
Supplement: S1 Appendix — (DOCX) [file pone.0306772.s001.docx]

S1 Appendix. Rating manual for patient motivation

# **General introduction**

This rating captures the concept of motivation of a study sample to be interested to seek a specific treatment and to become part of a randomized controlled trial. A higher motivation at baseline might be related to higher outcome expectations about the specific treatment and higher engagement during the treatment. This rating tool was developed for mHealth studies in which participants have an active role in the treatment and motivation is key. The rating is based on study level information, this means, information which is available across all participants of a study will be considered. For the rating any kind of information (main publication, protocol, preliminary study, etc.) should be considered.

We identified three indicators as relevant to capture participants’ motivation at baseline. Each of these indicators must be rated and has three levels: high motivation, moderate motivation, and low motivation. If no sufficient information is presented, the indicator is rated as low motivation with an additional coding as “unclear”. The additional coding is necessary to identify studies with unclear information after the rating.

If contradicting information is presented for one indicator and more than one level of motivation could be rated, the reviewer has to outweigh the relevance of the information. In general, the rating of a higher level of motivation should be preferred over a lower level of motivation.

Finally, the rating patterns from the three indicators will be considered according to the rating tree diagram to generate a final rating from high, to moderate and low motivation.

The raters must record their certainty of rating for each indicator independently. The certainty of the rating has three levels: high certainty, moderate certainty, and low certainty. Discrepancies of the rating will be solved by consensus between the raters after the initial rating. Making notes is essential during the coding. This may help to get to a better consensus later. An extra field for notes is present on the rating form.

# **Description of the three indicators**

## Indicator 1. Study team actively selects or enhances the motivation of the potential study participants

*Description: the study recruited predominately participants with high motivation, or excluded participants with low motivation during the screening, or used strategy to increase participants’ motivation before the randomization.*

*Relevant information is the description of* ***selection processes*** *which goes along with participants’ motivation. Other relevant information is the* ***communication*** *between the study team and the participant, which may increase the motivation to become part of the study. It is assumed that the selection of highly motivated participants or a specific communication goes along with a high motivation of the study participants for a specific intervention.*

### Rate high motivation

- The study recruited participants with high motivation about the intervention

Example:

1. The raters judge that from the recruitment strategy or baseline data that participants indicated a strong interest to participate, or have high expectations towards the intervention.

- The study excluded participants with low motivation before the randomization

Examples:

1. "Exclusion criteria... refusal to participate in follow-up assessments"
2. “The exclusion criteria consisted of failing to regularly participate in the educational therapeutic program”
3. “Patients were excluded if …had no access to the telecommunication environment during their BPSS app use, or were extremely unskilled in operating the BPSS app”

- Study participants have strong beliefs towards the importance of health either in general or concerning their specific clinical situation

Examples:

- - - 1. General: The raters judge that from the recruitment strategy or baseline data that participants have high self-control or high self-efficacy towards health.
      2. Specific: The study participants have high knowledge, health literacy etc. about their disease.
- Study participants are open about mHealth interventions in general or for investigated intervention

Examples:

1. The raters judge that from the recruitment strategy or baseline data that participants have high digital health literacy.
2. The raters judge that from the recruitment strategy or baseline data that more than 40% of participants were highly educated (i.e., university education level and / or above).

- The study team involved participating patients as stakeholders in trial design, trial intervention or setup of the study
- During the recruitment / screening phase, the study team provided consultant or education (that beyond the standard introduction of the trial) to enhance participants’ motivation to become enrolled in the study
- During the recruitment / screening phase, the study team advertised the study about the overall clinical benefit (strong marketing that beyond the standard introduction of the trial)
- During the recruitment / screening phase, the study team advertised the study with the achieved earlier success in the preliminary feasibility phase or in the pilot phase

### Rate moderate motivation

- Study participants indicated the interest to participate

Examples:

1. “Contact was made with individuals interested in voluntarily participating in the study. A member of the research team contacted each interested participant and explained the objectives and methods of the study, and received written consent from each patient”
2. “Interested participants can leave their contact details at the website. The research team will send them a screening questionnaire to assess eligibility”

- The study recruited the participants via a “normal / standard” approach, without any specific pre-selection criteria based on the motivation

Examples:

1. “We targeted for participation patients with diabetes who had inadequate glucose control while taking oral diabetes medications, with or without the concomitant use of insulin”
2. “For inclusion in the study, participants had to understand the study objectives, voluntarily agree to participate, and sign a written consent form”

- During the recruitment / screening phase, the study team specified that there are no financial compensation nor incentives of any kind

### Rate low motivation

- The study recruited participants with rather low motivation

Examples:

1. Participants were suffering from certain clinical conditions or certain routine medication which are strongly associated with decreased energy or motivation to actively engage in self-care activities.
2. The raters judge that from the recruitment strategy or baseline data that participants have weak beliefs towards the importance of health.
3. The raters judge that from the recruitment strategy or baseline data that participants have low digital health literacy.

### Rate low motivation with an additional coding of unclear

- No information
- Irrelevant information

Examples:

1. "patients were excluded if they were likely to change medication regimen during the course of the study"
2. “patients were excluded from the analysis if they are lost to follow-up”
3. The study team offer financial compensation or incentives to participants for participation.
4. Patients suffer from mental health problems (e.g., depression).

- Insufficient information for raters to make judgement

Example:

1. The study sample included a broad range of participants.

## Indicator 2. Participants active engagement before allocation

*Description: Participants have to invest a large amount of effort before the randomization to become a participant of the study.*

*Relevant information is the description about the effort, duration, time etc. of the screening and enrolment process, which reflects* ***the effort of the participants in advance (before randomization)****. It is assumed that a high effort of the participants goes along with a high motivation of the study participants for a specific intervention.*

### Rate high motivation

- Participants need to complete complicated steps before allocation which requires the participants to take actions, and the whole process is rather time-consuming

Examples:

1. “Eligible participants who passed the telephone screening were invited for a screening baseline visit that included a physical exam (weight, height, BMI, waist and hip circumference, and blood pressure), fasting blood draw (eg, fasting plasma glucose and hemoglobin A1c), and questionnaires. Those who passed the screening baseline visit and fasting blood draw received a Fitbit Zip accelerometer and Fitbit app with diary with training and were then enrolled in a 14-day run-in period… The run-in period was designed to screen out potential noncompliant participants… Those who complied at least 70% of the time with the run-in requirements demonstrated readiness for behaviors change and were enrolled and randomized into the study.”
2. "We recruited community-dwelling women through our website, www.econtinence.se, where they filled out a questionnaire to determine whether they met the study criteria… Women who met the inclusion criteria received a letter with informed consent and a 2-day leakage diary including a maximum voiding volume… After returning the informed consent and leakage diary, participants answered a web-based questionnaire that recorded background characteristics and lifestyle. They also completed two validated questionnaire"
3. “These veterans were solicited via letter and telephone. They asked to enroll by responding affirmatively over the phone or returned a card via mail. Those patients who subsequently provided an e-mail address, completed an online survey, submitted an HbA1c home test kit (Home Access Inc., Hoffman Estates, IL), and had a home-tested HbA1c value of $58 mmol/mol were included in the study.”
4. In addition to the eligibility screening questionnaire and the socio-demographic questionnaire, the other baseline questionnaires have more than 80 items in total for participants to complete

- Participants need to complete the informed consent via a rather complicated procedure

### Rate moderate motivation

- Participants need to complete simple steps before allocation, and the procedure is rather convenient or smooth for participants

Examples:

1. “Eligible participants receive an information letter and consent form with a return envelope. After receiving the signed consent form, participants are invited to fill out an online screening battery”
2. In addition to the eligibility screening questionnaire and the socio-demographic questionnaire, the other baseline questionnaires have 20 - 80 items in total for participants to complete.

### Rate low motivation

- Participants didn’t need to actively do much things before allocation

Examples:

1. The recruitment site or family caregiver contributed major support for participants’ enrolment, and participants themselves didn’t need to do much.
2. In addition to the eligibility screening questionnaire and the socio-demographic questionnaire, the other baseline questionnaires have less than 20 items in total for participants to complete.

### Rate low motivation with an additional coding of unclear

- No information
- Irrelevant information

Examples:

1. "Exclusion criteria... patients… failing to provide consent"
2. "Patients were included if... adequate familiarity in the use of mobile phones according to the physician judgment"

## Indicator 3. Potential trust between participant and the person / institution referring to the study

*Description: The recruitment is conducted via a person / institution the potential participant is attached to.*

*Relevant information is the description about* ***the potential trust and bond*** *between the participant and the referring person or institution. It is assumed that a strong trust and bond goes along with high motivation of the study participants for a specific intervention.*

### Rate high motivation

- Participants (more than 50%) were recruited or referred to the study by their treating physician or from their treating institution

Examples:

1. “After introduction from clinical staff, eligible patients will be approached by a member of the research team who will explain the study and provide the information sheet”
2. “The majority of the study sample were recruited by medical center staff (102/172; 59%), and the remainder of the sample through social media (70/172; 41%)”

- Participants were recruited from the institution where they were previously treated or they scheduled to receive treatment, and the study was also conducted in this institution

Example:

- - - 1. “Inclusion criterial… treated with chemotherapy at the study sites”

### Rate moderate motivation

- Participants were recruited or referred by a mix of approaches

Example:

1. Patients were recruited or referred by their treating physician, public flyers, and social media advertisement, and none of these single methods recruited more than 50% of participants

- Participants were recruited via patients communities

Examples:

1. Disease education in patient community
2. Advertisement of the study in patient community
3. Word-of-mouth between patients (e.g., self-help group)

### Rate low motivation

- Participants were approached and recruited via a database or health care records, and the raters judge that there is lack of trust due to yet non-existent relationship with the study team

Examples:

1. "The electronic health records were utilized to screen patients with either heart disease or diabetes mellitus type 2... Of these patients, 499 heart disease patients and 500 diabetes patients were randomly selected and received invitation letters in October 2010… In total, invitation letters were sent to 2084 patients, of which 28.02% (584) agreed to participate"
2. "Invitation letters were sent to 337 patients who were screened from the electronic health record system at the Sipoo,Finland, Community Health Centre. In total, 237 patients did not respond to the invitation letter or declined to participate. Of the 100 respondents who indicated an interest in the study, 35 were excluded as ineligible, and nine patients subsequently declined to participate. "
3. “Eligible patients were identified through the medical appointment lists at the two clinics. During the patients’ first visit at the oncology clinic, the patients received written information about the study from the assigned oncology contact nurse or physician. The patients were then contacted by the researcher and asked about participation.”
4. “Research staff identified potentially eligible patients by querying the EHR. Following written or verbal approval from the patients’ oncologists, staff approached patients who met initial inclusion criteria at their upcoming clinic visit.”

- Participants were recruited via media of all sorts with mass distribution

Examples:

1. Social media (e.g., Facebook ads)
2. Print media (e.g., flyers, brochures, newspaper)
3. Online / digital content (e.g., a study website or search engine ads)
4. Television / radio / press release

### Rate low motivation with an additional coding of unclear

- It is unclear how were the patients recruited or being approached

Example:

- - - 1. “Patients with pathologically proven, clinical stage IV breast cancer were enrolled in this study at Chung-Ang University Hospital, Korea, from September 2013 to September 2014.”
