# Supplementary material for: Patient motivation as a predictor of digital health intervention effects: A meta-epidemiological study of cancer trials
Source: PLoS One. 2024 Jul 8;19(7):e0306772. doi: 10.1371/journal.pone.0306772 (PMC11230537; doi:10.1371/journal.pone.0306772)
Supplement: S2 Appendix — (DOCX) [file pone.0306772.s002.docx]

**S3 Appendix. Training records of the raters**

|  |  |  |  |  |  |  |  |  |  |
| --- | --- | --- | --- | --- | --- | --- | --- | --- | --- |

Participants

EJ Elena Jimenez Tejero

ES Elena Stallings

JB Jürgen Barth

YY Yuqian Yan

|  |  |  |  |  |  |  |  |  |  |
| --- | --- | --- | --- | --- | --- | --- | --- | --- | --- |

| **Date** | **Participants** | **Topic** | **Actions and responsible persons** |
| --- | --- | --- | --- |
| March 22, 2023 | YY, JB, ES, EJ | Kickoff meeting | Agenda (45 minutes):   1. All participants introduced their academic backgrounds; 2. JB and YY introduced about the Motivation Meta-Epidemiological Study, and the upcoming rating tasks for all participants; 3. ES and EJ were invited to read the rating manual before the rating training. |
| March 30, 2023 | YY, JB, ES, EJ | Rating training | Agenda (2 hours):   1. Questions and answers session; 2. JB and YY lead the participants to read through the rating manual while highlighting the important aspects of each indicator; 3. JB and YY demonstrated the rating procedure on a pre-designed rating form; 4. JB and YY explained how to rate for each indicators with examples based on three studies; 5. ES and EJ conducted the rating for two studies independently and followed by a discussion among all participants. |
| March 31 – April 11, 2023 | YY, JB, ES, EJ | Pilot testing | All participants independently rated five new studies. During the rating they were blinded from other participants’ ratings. |
| April 13, 2023 | YY, JB, ES, EJ | Rating consensus | Agenda (2 hours):   1. All participants discussed about the questions they encountered during the pilot testing, and discussed about their differences in rating; 2. All participants agreed to modify some of the rating rules in the rating manual, mainly:  - Indicator 1 - We no longer consider recruitment with “targeting strategy” and participants with mental health problems (e.g., depression) as relevant for rating; - We decided to rate moderate motivation when the study recruited participants with “normal / standard” approach (e.g., no specific pre-selection based on the motivation) - Indicator 2 - We specified the judgment for the amount of effort: high effort (high motivation) would require multiple steps that might be time-consuming; moderate effort (moderate motivation) include rather convenient or smooth procedures that could be considered as “standard” effort for a trial |
