# Supplementary material for: Patient motivation as a predictor of digital health intervention effects: A meta-epidemiological study of cancer trials
Source: PLoS One. 2024 Jul 8;19(7):e0306772. doi: 10.1371/journal.pone.0306772 (PMC11230537; doi:10.1371/journal.pone.0306772)
Supplement: S3 Appendix — (DOCX) [file pone.0306772.s003.docx]

**S4 Appendix. Included studies**

1. Børøsund E, Ehlers SL, Clark MM, Andrykowski MA, Cvancarova Småstuen M, Solberg Nes L. Digital stress management in cancer: testing StressProffen in a 12-month randomized controlled trial. Cancer 2022 Apr 01;128(7):1503-1512.
2. Çınar D, Karadakovan A, Erdoğan AP. Effect of mobile phone app-based training on the quality of life for women with breast cancer. Eur J Oncol Nurs 2021 Jun;52:101960.
3. Fjell M, Langius-Eklöf A, Nilsson M, Wengström Y, Sundberg K. Reduced symptom burden with the support of an interactive app during neoadjuvant chemotherapy for breast cancer - a randomized controlled trial. Breast 2020 Jun;51:85-93.
4. Ghanbari E, Yektatalab S, Mehrabi M. Effects of psychoeducational interventions using mobile apps and mobile-based online group discussions on anxiety and self-esteem in women with breast cancer: randomized controlled trial. JMIR Mhealth Uhealth 2021 May 18;9(5):e19262.
5. Greer J, Jacobs J, Pensak N, MacDonald J, Fuh C, Perez G, et al. Randomized trial of a tailored cognitive-behavioral therapy mobile application for anxiety in patients with incurable cancer. Oncologist 2019 Aug;24(8):1111-1120.
6. Greer J, Jacobs JM, Pensak N, Nisotel LE, Fishbein J, MacDonald J, et al. Randomized trial of a smartphone mobile app to improve symptoms and adherence to oral therapy for cancer. J Natl Compr Canc Netw 2020 Feb;18(2):133-141.
7. Ham K, Chin S, Suh YJ, Rhee M, Yu E, Lee HJ, et al. Preliminary results from a randomized controlled study for an app-based cognitive behavioral therapy program for depression and anxiety in cancer patients. Front Psychol 2019 Jul 25;10:1592.
8. Handa S, Okuyama H, Yamamoto H, Nakamura S, Kato Y. Effectiveness of a smartphone application as a support tool for patients undergoing breast cancer chemotherapy: a randomized controlled trial. Clin Breast Cancer 2020 Jun;20(3):201-208.
9. Karaaslan-Eşer A, Ayaz-Alkaya S. The effect of a mobile application on treatment adherence and symptom management in patients using oral anticancer agents: a randomized controlled trial. Eur J Oncol Nurs 2021 Jun;52:101969.
10. Kim HJ, Kim SM, Shin H, Jang J, Kim YI, Han DH. A mobile game for patients with breast cancer for chemotherapy self-management and quality-of-life improvement: randomized controlled trial. J Med Internet Res 2018 Oct 29;20(10):e273.
11. Kubo A, Kurtovich E, McGinnis M, Aghaee S, Altschuler A, Quesenberry C, et al. Pilot pragmatic randomized trial of mHealth mindfulness-based intervention for advanced cancer patients and their informal caregivers. Psychooncology 2020 Sep 26 (forthcoming).
12. Park HR, Kang HS, Kim SH, Singh-Carlson S. Effect of a smart pill bottle reminder intervention on medication adherence, self-efficacy, and depression in breast cancer survivors. Cancer Nurs 2021 Oct 12;45(6):E874-E882.
13. Spahrkäs SS, Looijmans A, Sanderman R, Hagedoorn M. Beating cancer-related fatigue with the Untire mobile app: results from a waiting-list randomized controlled trial. Psychooncology 2020 Nov 11;29(11):1823-1834.
14. Sui Y, Wang T, Wang X. The impact of WeChat app-based education and rehabilitation program on anxiety, depression, quality of life, loss of follow-up and survival in non-small cell lung cancer patients who underwent surgical resection. Eur J Oncol Nurs 2020 Apr;45:101707.
15. Zhou K, Li J, Li X. Effects of cyclic adjustment training delivered via a mobile device on psychological resilience, depression, and anxiety in Chinese post-surgical breast cancer patients. Breast Cancer Res Treat 2019 Nov 24;178(1):95-103.
16. Zhu J, Ebert L, Liu X, Wei D, Chan SW. Mobile breast cancer e-support program for Chinese women with breast cancer undergoing chemotherapy (part 2): multicenter randomized controlled trial. JMIR Mhealth Uhealth 2018 Apr 30;6(4):e104.
17. Di R, Li G. Use of a smartphone medical app improves complications and quality of life in patients with nasopharyngeal carcinoma who underwent radiotherapy and chemotherapy. Med Sci Monit 2018 Sep 04;24:6151-6156.
18. Dong X, Yi X, Gao D, Gao Z, Huang S, Chao M, et al. The effects of the combined exercise intervention based on internet and social media software (CEIBISMS) on quality of life, muscle strength and cardiorespiratory capacity in Chinese postoperative breast cancer patients: a randomized controlled trial. Health Qual Life Outcomes 2019 Jun 26;17(1):109.
19. Hou I, Lin H, Shen S, Chang K, Tai H, Tsai A, et al. Quality of life of women after a first diagnosis of breast cancer using a self-management support mHealth app in Taiwan: randomized controlled trial. JMIR Mhealth Uhealth 2020 Mar 04;8(3):e17084.
20. Rosen KD, Paniagua SM, Kazanis W, Jones S, Potter JS. Quality of life among women diagnosed with breast Cancer: a randomized waitlist controlled trial of commercially available mobile app-delivered mindfulness training. Psychooncology 2018 Aug 01;27(8):2023-2030.
21. Zha G. Effect of WeChat continuity of care on psychological stress, self-care ability and quality of life of chemotherapy patients after radical breast cancer surgery. J Practical Cardiopulmonary Vascular Disease 2020 Jul 30:148-150.
22. Absolom K, Warrington L, Hudson E, Hewison J, Morris C, Holch P, et al. Phase III randomized controlled trial of eRAPID: eHealth intervention during chemotherapy. J Clin Oncol 2021 Mar 01;39(7):734-747.
23. Berg CJ, Vanderpool RC, Getachew B, Payne JB, Johnson MF, Sandridge Y, et al. A hope-based intervention to address disrupted goal pursuits and quality of life among young adult cancer survivors. J Cancer Educ 2020 Dec 11;35(6):1158-1169.
24. Chen K, Yao F, Chen X, Lin Y, Kang M. Effectiveness of telerehabilitation on short-term quality of life of patients after esophageal cancer surgery during COVID-19: a single-center, randomized, controlled study. J Gastrointest Oncol 2021 Aug;12(4):1255-1264.
25. Huggins CE, Hanna L, Furness K, Silvers MA, Savva J, Frawley H, et al. Effect of early and intensive telephone or electronic nutrition counselling delivered to people with upper gastrointestinal cancer on quality of life: a three-arm randomised controlled trial. Nutrients 2022 Aug 07;14(15):3234.
26. Maguire R, McCann L, Kotronoulas G, Kearney N, Ream E, Armes J, et al. Real time remote symptom monitoring during chemotherapy for cancer: European multicentre randomised controlled trial (eSMART). BMJ 2021 Jul 21;374:n1647.
27. Seib C, Anderson D, McGuire A, Porter-Steele J, McDonald N, Balaam S, et al. Improving health-related quality of life in women with breast, blood, and gynaecological cancer with an eHealth-enabled 12-week lifestyle intervention: the women's wellness after Cancer program randomised controlled trial. BMC Cancer 2022 Jul 08;22(1):747.
