# Supplementary material for: Patient motivation as a predictor of digital health intervention effects: A meta-epidemiological study of cancer trials
Source: PLoS One. 2024 Jul 8;19(7):e0306772. doi: 10.1371/journal.pone.0306772 (PMC11230537; doi:10.1371/journal.pone.0306772)
Supplement: S4 Appendix — (DOCX) [file pone.0306772.s004.docx]

S5 Appendix. Certainty levels of 4 raters per studies (number of rated studies = 27)

| **Study ID** | **Rater** | **Indicator 1** | **Indicator 2** | **Indicator 3** |
| --- | --- | --- | --- | --- |
| Absolom 2021 | Rater 1 | Very certain | Very certain | Moderate certain |
|  | Rater 2 | Very certain | Very certain | Very certain |
|  | Rater 3 | Very certain | Very certain | Very certain |
|  | Rater 4 | Very certain | Very certain | Very certain |
| Berg 2019 | Rater 1 | Moderate certain | Very certain | Very certain |
|  | Rater 2 | Very certain | Very certain | Moderate certain |
|  | Rater 3 | Moderate certain | Very certain | Very certain |
|  | Rater 4 | Very certain | Very certain | Very certain |
| Børøsund 2022 | Rater 1 | Very certain | Very certain | Moderate certain |
|  | Rater 2 | Very certain | Very certain | Very certain |
|  | Rater 3 | Very certain | Moderate certain | Very certain |
|  | Rater 4 | Very certain | Very certain | Moderate certain |
| Chen 2021 | Rater 1 | Moderate certain | Very certain | Unclear |
|  | Rater 2 | Unclear | Unclear | Very certain |
|  | Rater 3 | Not certain | Not certain | Very certain |
|  | Rater 4 | Very certain | Unclear | Very certain |
| Çınar 2021 | Rater 1 | Moderate certain | Moderate certain | Unclear |
|  | Rater 2 | Unclear | Unclear | Very certain |
|  | Rater 3 | Unclear | Unclear | Very certain |
|  | Rater 4 | Unclear | Unclear | Unclear |
| Di 2018 | Rater 1 | Moderate certain | Moderate certain | Not certain |
|  | Rater 2 | Unclear | Unclear | Very certain |
|  | Rater 3 | Very certain |  | Very certain |
|  | Rater 4 | Very certain | Unclear | Very certain |
| Dong 2019 | Rater 1 | Moderate certain | Moderate certain | Unclear |
|  | Rater 2 | Unclear | Unclear | Not certain |
|  | Rater 3 | Not certain | Not certain | Very certain |
|  | Rater 4 | Very certain | Unclear | Not certain |
| Fjell 2020 | Rater 1 | Not certain | Unclear | Unclear |
|  | Rater 2 | Very certain | Very certain | Very certain |
|  | Rater 3 | Moderate certain | Moderate certain | Very certain |
|  | Rater 4 | Very certain | Very certain | Very certain |
| Foley 2016 | Rater 1 | Moderate certain | Unclear | Unclear |
|  | Rater 2 | Unclear | Unclear | Not certain |
|  | Rater 3 | Not certain | Not certain | Unclear |
|  | Rater 4 | Very certain | Unclear | Moderate certain |
| Ghanbari 2021 | Rater 1 | Moderate certain | Very certain | Very certain |
|  | Rater 2 | Very certain | Very certain | Very certain |
|  | Rater 3 | Moderate certain | Unclear | Very certain |
|  | Rater 4 | Very certain | Very certain | Very certain |
| Greer 2019 | Rater 1 | Very certain | Very certain | Moderate certain |
|  | Rater 2 | Very certain | Very certain | Very certain |
|  | Rater 3 | Moderate certain | Moderate certain | Moderate certain |
|  | Rater 4 | Not certain | Very certain | Very certain |
| Greer 2020 | Rater 1 | Very certain | Very certain | Moderate certain |
|  | Rater 2 | Very certain | Moderate certain | Very certain |
|  | Rater 3 | Not certain | Not certain | Very certain |
|  | Rater 4 | Not certain | Moderate certain | Very certain |
| Ham 2019 | Rater 1 | Very certain | Moderate certain | Very certain |
|  | Rater 2 | Very certain | Moderate certain | Very certain |
|  | Rater 3 | Very certain | Very certain | Unclear |
|  | Rater 4 | Very certain | Not certain | Very certain |
| Handa 2020 | Rater 1 | Moderate certain | Very certain | Very certain |
|  | Rater 2 | Very certain | Moderate certain | Very certain |
|  | Rater 3 | Moderate certain | Not certain | Very certain |
|  | Rater 4 | Not certain | Moderate certain | Very certain |
| Hou 2020 | Rater 1 | Very certain | Moderate certain | Very certain |
|  | Rater 2 | Unclear | Moderate certain | Very certain |
|  | Rater 3 | Moderate certain | Moderate certain | Very certain |
|  | Rater 4 | Not certain | Not certain | Very certain |
| Huggins 2022 | Rater 1 | Very certain | Moderate certain | Moderate certain |
|  | Rater 2 | Moderate certain | Unclear | Very certain |
|  | Rater 3 |  | Very certain | Not certain |
|  | Rater 4 | Very certain | Not certain | Moderate certain |
| Karaaslan-Eşer 2021 | Rater 1 | Moderate certain | Moderate certain | Unclear |
|  | Rater 2 | Unclear | Unclear | Very certain |
|  | Rater 3 | Unclear | Unclear |  |
|  | Rater 4 | Not certain | Very certain | Very certain |
| Kim 2018 | Rater 1 | Moderate certain | Moderate certain | Unclear |
|  | Rater 2 | Unclear | Unclear | Very certain |
|  | Rater 3 |  |  |  |
|  | Rater 4 | Very certain | Unclear | Unclear |
| Kubo 2020 | Rater 1 | Very certain | Moderate certain | Very certain |
|  | Rater 2 | Unclear | Not certain | Moderate certain |
|  | Rater 3 | Not certain | Not certain | Not certain |
|  | Rater 4 | Very certain | Unclear | Very certain |
| Maguire 2021 | Rater 1 | Very certain | Moderate certain | Unclear |
|  | Rater 2 | Very certain | Not certain | Moderate certain |
|  | Rater 3 | Not certain | Moderate certain | Very certain |
|  | Rater 4 | Very certain | Moderate certain | Very certain |
| Park 2021 | Rater 1 | Moderate certain | Moderate certain |  |
|  | Rater 2 | Not certain | Very certain | Very certain |
|  | Rater 3 | Moderate certain | Very certain | Very certain |
|  | Rater 4 | Very certain | Very certain | Not certain |
| Rosen 2018 | Rater 1 | Very certain | Very certain | Very certain |
|  | Rater 2 | Moderate certain | Very certain | Very certain |
|  | Rater 3 | Very certain | Moderate certain | Very certain |
|  | Rater 4 | Very certain | Moderate certain | Moderate certain |
| Seib 2022 | Rater 1 | Very certain | Very certain | Very certain |
|  | Rater 2 | Very certain | Unclear | Not certain |
|  | Rater 3 | Not certain | Not certain | Very certain |
|  | Rater 4 | Very certain | Moderate certain | Very certain |
| Spahrkäs 2020 | Rater 1 | Very certain | Very certain | Moderate certain |
|  | Rater 2 | Moderate certain | Moderate certain | Very certain |
|  | Rater 3 | Very certain | Very certain | Very certain |
|  | Rater 4 | Very certain | Moderate certain | Very certain |
| Sui 2020 | Rater 1 | Moderate certain | Moderate certain | Unclear |
|  | Rater 2 | Unclear | Unclear |  |
|  | Rater 3 |  |  | Very certain |
|  | Rater 4 | Moderate certain | Moderate certain | Very certain |
| Zhou 2019 | Rater 1 | Moderate certain | Moderate certain | Unclear |
|  | Rater 2 | Unclear | Unclear | Moderate certain |
|  | Rater 3 | Not certain | Moderate certain |  |
|  | Rater 4 | Very certain | Moderate certain | Unclear |
| Zhu 2018 | Rater 1 | Moderate certain | Very certain | Moderate certain |
|  | Rater 2 | Unclear | Unclear | Very certain |
|  | Rater 3 | Very certain | Very certain | Very certain |
|  | Rater 4 | Very certain | Very certain | Very certain |

***Notes****:* *Blank cell means that that the rater did not specify the level of certainty (missing data).*
