# Supplementary material for: Patient motivation as a predictor of digital health intervention effects: A meta-epidemiological study of cancer trials
Source: PLoS One. 2024 Jul 8;19(7):e0306772. doi: 10.1371/journal.pone.0306772 (PMC11230537; doi:10.1371/journal.pone.0306772)
Supplement: S5 Appendix — (DOCX) [file pone.0306772.s005.docx]

S6 Appendix. Summary of certainty levels per raters (number of rated studies = 27)

| **Rater** | **Level of Certainty** | **No. of Studies (%)** | | |
| --- | --- | --- | --- | --- |
|  |  | **Indicator 1** | **Indicator 2** | **Indicator 3** |
| **Rater 1** | Very certain | 12 (44) | 12 (44) | 8 (30) |
|  | Moderate certain | 14 (52) | 13 (48) | 7 (26) |
|  | Not certain | 1 (4) | 0 | 1 (4) |
|  | Unclear ^a^ | 0 | 2 (7) | 10 (37) |
|  | No response ^b^ | 0 | 0 | 1 (4) |
| **Rater 2** | Very certain | 11 (41) | 8 (30) | 19 (70) |
|  | Moderate certain | 3 (11) | 5 (19) | 4 (15) |
|  | Not certain | 1 (4) | 2 (7) | 3 (11) |
|  | Unclear ^a^ | 12 (44) | 12 (44) | 0 |
|  | No response ^b^ | 0 | 0 | 1 (4) |
| **Rater 3** | Very certain | 7 (26) | 7 (26) | 19 (70) |
|  | Moderate certain | 7 (26) | 7 (26) | 1 (4) |
|  | Not certain | 8 (30) | 7 (26) | 2 (7) |
|  | Unclear ^a^ | 2 (7) | 3 (11) | 2 (7) |
|  | No response ^b^ | 3 (11) | 3 (11) | 3 (11) |
| **Rater 4** | Very certain | 20 (74) | 9 (33) | 18 (67) |
|  | Moderate certain | 1 (4) | 8 (30) | 4 (15) |
|  | Not certain | 5 (19) | 3 (11) | 2 (7) |
|  | Unclear ^a^ | 1 (4) | 7 (26) | 3 (11) |
|  | No response ^b^ | 0 | 0 | 0 |
|  |  |  |  |  |
| ***Notes:*** ***a****. Unclear means that the rater did not specify the level of certainty due to insufficient information provided for the rating (in this case, the indicator is rated as low motivation);* ***b****. No response means that that the rater did not specify the level of certainty (missing data).* | | | | |
