# Supplementary material for: Patient motivation as a predictor of digital health intervention effects: A meta-epidemiological study of cancer trials
Source: PLoS One. 2024 Jul 8;19(7):e0306772. doi: 10.1371/journal.pone.0306772 (PMC11230537; doi:10.1371/journal.pone.0306772)
Supplement: S6 Appendix — (DOCX) [file pone.0306772.s006.docx]

**S7 Appendix. ICC based on five ratings (four raters and consensus) for the overall rating and each indicator**

| **Inter-rater reliability** | **ICC** | **95% CI** | | ***P*** |
| --- | --- | --- | --- | --- |
|  |  | **LL** | **UL** |  |
| Overall motivation | 0.62 | 0.32 | 0.81 | < 0.001 |
| Indicator 1 (expectation) | 0.68 | 0.43 | 0.84 | < 0.001 |
| Indicator 2 (effort) | 0.59 | 0.27 | 0.79 | 0.001 |
| Indicator 3 (bond) | 0.60 | 0.29 | 0.80 | < 0.001 |

**Abbreviations**: CI, confidence interval; LL, lower limit; UL, upper limit.
