# Supplementary material for: Patient motivation as a predictor of digital health intervention effects: A meta-epidemiological study of cancer trials
Source: PLoS One. 2024 Jul 8;19(7):e0306772. doi: 10.1371/journal.pone.0306772 (PMC11230537; doi:10.1371/journal.pone.0306772)
Supplement: S7 Appendix — (DOCX) [file pone.0306772.s007.docx]

**S12 Appendix. Sensitivity analysis: Equal weighting of indicators for overall motivation**

**Table. Meta-regression and subgroup analysis on quality of life**

| **Characteristics** | **Studies** | **SMD** | **95% CI** | | **Heterogeneity** | | **Meta-regression** |
| --- | --- | --- | --- | --- | --- | --- | --- |
|  |  |  | **LL** | **UL** | ***I^2^* (%)** | ***P*** | ***P*** |
| **All studies** | 22 | 0.31 | 0.17 | 0.46 | 64 | < 0.001 |  |
| **Overall motivation** |  |  |  |  |  |  | 0.103 |
| High motivation | 9 | 0.32 | 0.21 | 0.42 | 15 | 0.312 |  |
| Moderate motivation | 10 | 0.42 | 0.20 | 0.64 | 63 | 0.004 |  |
| Low motivation | 3 | -0.04 | -1.51 | 1.43 | 85 | 0.002 |  |
| **Indicator 1 (expectation)** |  |  |  |  |  |  | 0.886 |
| High motivation | 13 | 0.32 | 0.22 | 0.41 | 34 | 0.112 |  |
| Moderate motivation | 9 | 0.33 | -0.03 | 0.70 | 80 | < 0.001 |  |
| Low motivation | 0 |  |  |  |  |  |  |
| **Indicator 2 (effort)** |  |  |  |  |  |  | 0.872 |
| High motivation | 12 | 0.33 | 0.23 | 0.43 | 18 | 0.267 |  |
| Moderate motivation | 5 | 0.28 | 0.07 | 0.49 | 30 | 0.223 |  |
| Low motivation | 5 | 0.32 | -0.49 | 1.14 | 90 | < 0.001 |  |
| **Indicator 3 (bond)** |  |  |  |  |  |  | 0.367 |
| High motivation | 10 | 0.42 | 0.21 | 0.62 | 61 | 0.007 |  |
| Moderate motivation | 3 | 0.21 | -0.29 | 0.71 | 32 | 0.232 |  |
| Low motivation | 9 | 0.22 | -0.06 | 0.51 | 70 | < 0.001 |  |

Notes: SMD greater than zero indicates increase in quality of life favoring the mHealth app intervention group.

Abbreviations: SMD, standardized mean difference; CI, confidence interval; LL, lower limit; UL, upper limit.

**Table. Meta-regression and subgroup analysis on anxiety**

| **Characteristics** | **Studies** | **SMD** | **95% CI** | | **Heterogeneity** | | **Meta-regression** |
| --- | --- | --- | --- | --- | --- | --- | --- |
|  |  |  | **LL** | **UL** | ***I^2^* (%)** | ***P*** | ***P*** |
| **All studies** | 12 | -0.82 | -1.55 | -0.10 | 95 | < 0.001 |  |
| **Overall motivation** |  |  |  |  |  |  | 0.745 |
| High motivation | 6 | -0.52 | -0.99 | -0.06 | 82 | < 0.001 |  |
| Moderate motivation | 2 | -1.26 | -8.29 | 5.77 | 91 | < 0.001 |  |
| Low motivation | 4 | -1.02 | -4.20 | 2.17 | 98 | < 0.001 |  |
| **Indicator 1 (expectation)** |  |  |  |  |  |  | 0.534 |
| High motivation | 8 | -0.66 | -1.23 | -0.08 | 91 | < 0.001 |  |
| Moderate motivation | 4 | -1.15 | -4.15 | 1.85 | 98 | < 0.001 |  |
| Low motivation | 0 |  |  |  |  |  |  |
| **Indicator 2 (effort)** |  |  |  |  |  |  | 0.563 |
| High motivation | 5 | -0.46 | -0.99 | 0.06 | 79 | < 0.001 |  |
| Moderate motivation | 3 | -0.63 | -1.53 | 0.28 | 65 | 0.059 |  |
| Low motivation | 4 | -1.38 | -4.53 | 1.77 | 98 | < 0.001 |  |
| **Indicator 3 (bond)** |  |  |  |  |  |  | 0.891 |
| High motivation | 6 | -0.65 | -1.32 | 0.03 | 90 | < 0.001 |  |
| Moderate motivation | 1 | -1.21 | -1.80 | -0.63 | NA | NA |  |
| Low motivation | 5 | -0.96 | -3.11 | 1.20 | 98 | < 0.001 |  |

Notes: SMD less than zero indicates reduction in anxiety favoring the mHealth app intervention group. Abbreviations: SMD, standardized mean difference; CI, confidence interval; LL, lower limit; UL, upper limit; NA, not applicable.

**Table. Meta-regression and subgroup analysis on depression**

| **Characteristics** | **Studies** | **SMD** | **95% CI** | | **Heterogeneity** | | **Meta-regression** |
| --- | --- | --- | --- | --- | --- | --- | --- |
|  |  |  | **LL** | **UL** | ***I^2^* (%)** | ***P*** | ***P*** |
| **All studies** | 11 | -0.60 | -1.37 | 0.16 | 94 | < 0.001 |  |
| **Overall motivation** |  |  |  |  |  |  | 0.849 |
| High motivation | 5 | -0.41 | -0.86 | 0.04 | 65 | 0.022 |  |
| Moderate motivation | 2 | -0.44 | -1.21 | 0.33 | 0 | 0.713 |  |
| Low motivation | 4 | -0.91 | -4.11 | 2.29 | 98 | < 0.001 |  |
| **Indicator 1 (expectation)** |  |  |  |  |  |  | 0.567 |
| High motivation | 6 | -0.39 | -0.76 | -0.02 | 62 | 0.022 |  |
| Moderate motivation | 5 | -0.85 | -3.01 | 1.31 | 97 | < 0.001 |  |
| Low motivation | 0 |  |  |  |  |  |  |
| **Indicator 2 (effort)** |  |  |  |  |  |  | 0.549 |
| High motivation | 5 | -0.43 | -0.90 | 0.04 | 65 | 0.021 |  |
| Moderate motivation | 3 | -0.23 | -1.04 | 0.58 | 60 | 0.081 |  |
| Low motivation | 3 | -1.27 | -6.99 | 4.44 | 99 | < 0.001 |  |
| **Indicator 3 (bond)** |  |  |  |  |  |  | 0.814 |
| High motivation | 4 | -0.31 | -0.73 | 0.11 | 55 | 0.086 |  |
| Moderate motivation | 1 | -1.00 | -1.56 | -0.43 | NA | NA |  |
| Low motivation | 6 | -0.76 | -2.41 | 0.89 | 97 | < 0.001 |  |

Notes: SMD less than zero indicates reduction in depression favoring the mHealth app intervention group.

Abbreviations: SMD, standardized mean difference; CI, confidence interval; LL, lower limit; UL, upper limit; NA, not applicable.

**Table. Meta-regression and subgroup analysis on attrition**

| **Characteristics** | **Studies** | **RR** | **95% CI** | | **Heterogeneity** | | **Meta-regression** |
| --- | --- | --- | --- | --- | --- | --- | --- |
|  |  |  | **LL** | **UL** | ***I^2^* (%)** | ***P*** | ***P*** |
| **All studies** | 23 | 1.66 | 1.01 | 2.71 | 73 | < 0.001 |  |
| **Overall motivation** |  |  |  |  |  |  | 0.014 |
| High motivation | 10 | 1.01 | 0.52 | 1.96 | 61 | 0.006 |  |
| Moderate motivation | 8 | 4.36 | 1.99 | 9.53 | 25 | 0.227 |  |
| Low motivation | 5 | 1.28 | 0.49 | 3.32 | 1 | 0.400 |  |
| **Indicator 1 (expectation)** |  |  |  |  |  |  | 0.401 |
| High motivation | 14 | 1.98 | 0.96 | 4.06 | 80 | < 0.001 |  |
| Moderate motivation | 9 | 1.27 | 0.59 | 2.72 | 42 | 0.087 |  |
| Low motivation | 0 |  |  |  |  |  |  |
| **Indicator 2 (effort)** |  |  |  |  |  |  | 0.905 |
| High motivation | 12 | 1.81 | 0.86 | 3.81 | 64 | < 0.001 |  |
| Moderate motivation | 7 | 1.35 | 0.38 | 4.83 | 80 | < 0.001 |  |
| Low motivation | 4 | 1.45 | 0.41 | 5.10 | 15 | 0.317 |  |
| **Indicator 3 (bond)** |  |  |  |  |  |  | 0.115 |
| High motivation | 9 | 1.09 | 0.42 | 2.81 | 65 | 0.003 |  |
| Moderate motivation | 3 | 0.96 | 0.70 | 1.33 | 0 | 0.81 |  |
| Low motivation | 11 | 2.87 | 1.37 | 6.00 | 57 | 0.01 |  |

Notes: RR more than one indicates an increase in risk of dropouts among the mHealth intervention group as compared to the usual care group.

Abbreviations: RR, Risk ratio; CI, confidence interval; LL, lower limit; UL, upper limit.
