# Supplementary material for: Patient motivation as a predictor of digital health intervention effects: A meta-epidemiological study of cancer trials
Source: PLoS One. 2024 Jul 8;19(7):e0306772. doi: 10.1371/journal.pone.0306772 (PMC11230537; doi:10.1371/journal.pone.0306772)

Rating Tree Diagram

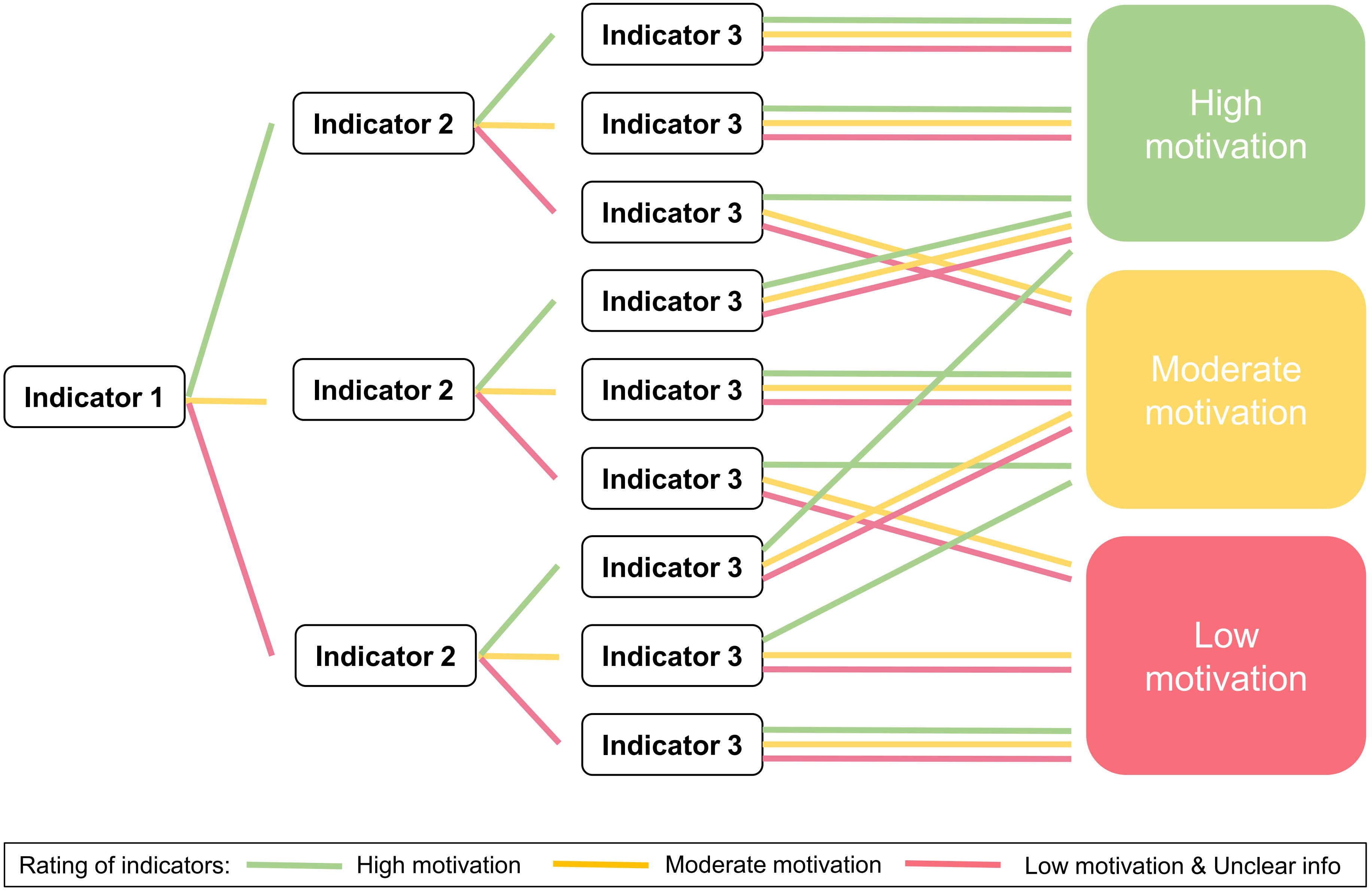

Supplement: S1 Fig — The rating tree is used to generate an overall rating of patient motivation at the study level based on the rating patterns from the three indicators. (PDF) [file pone.0306772.s008.pdf]
