# Supplementary figures and images for: Patient motivation as a predictor of digital health intervention effects: A meta-epidemiological study of cancer trials
Source: PLoS One. 2024 Jul 8;19(7):e0306772. doi: 10.1371/journal.pone.0306772 (PMC11230537; doi:10.1371/journal.pone.0306772)

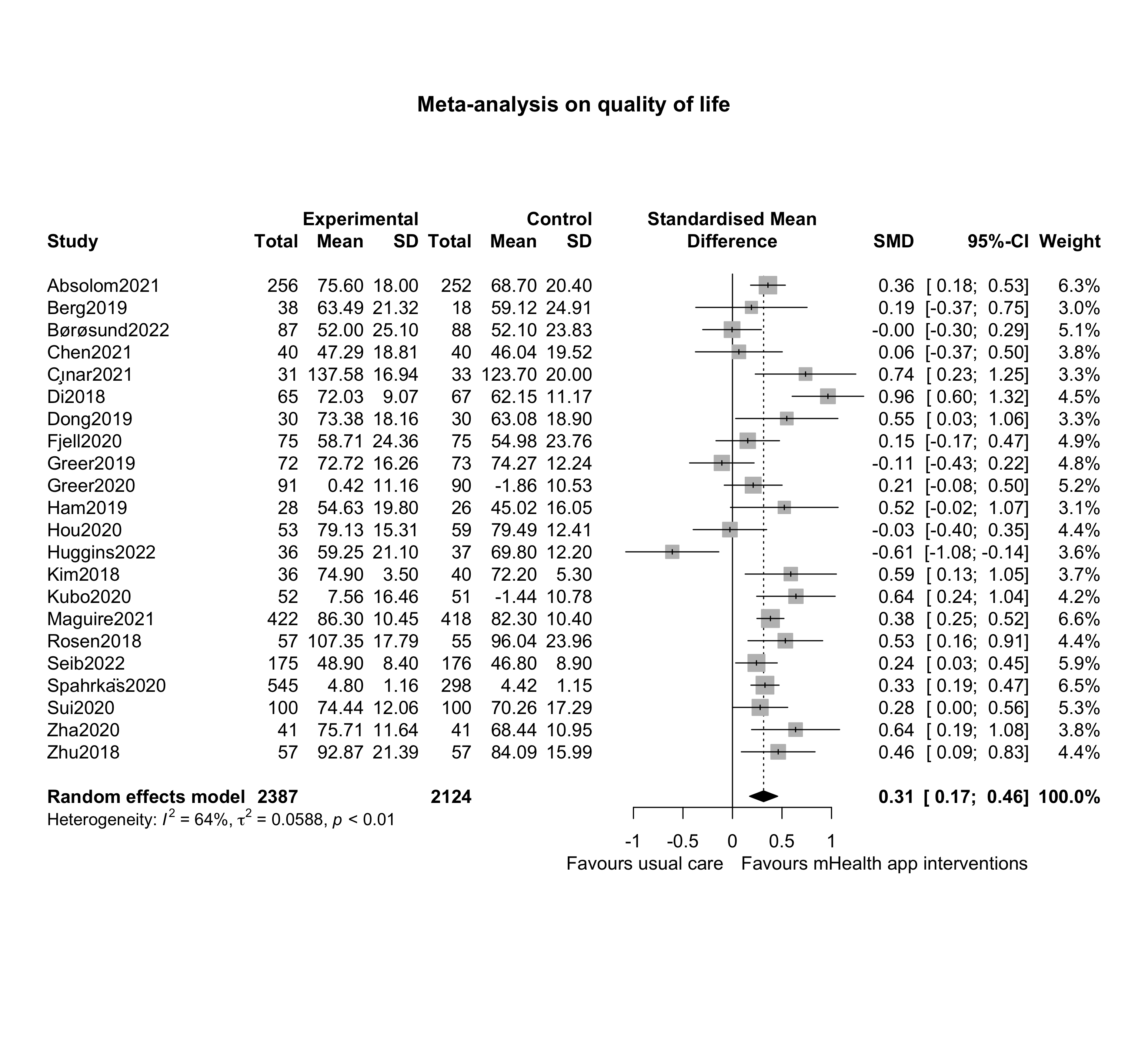

Supplement: S2 Fig — Meta-analysis on quality of life. (PNG) [file pone.0306772.s009.png]

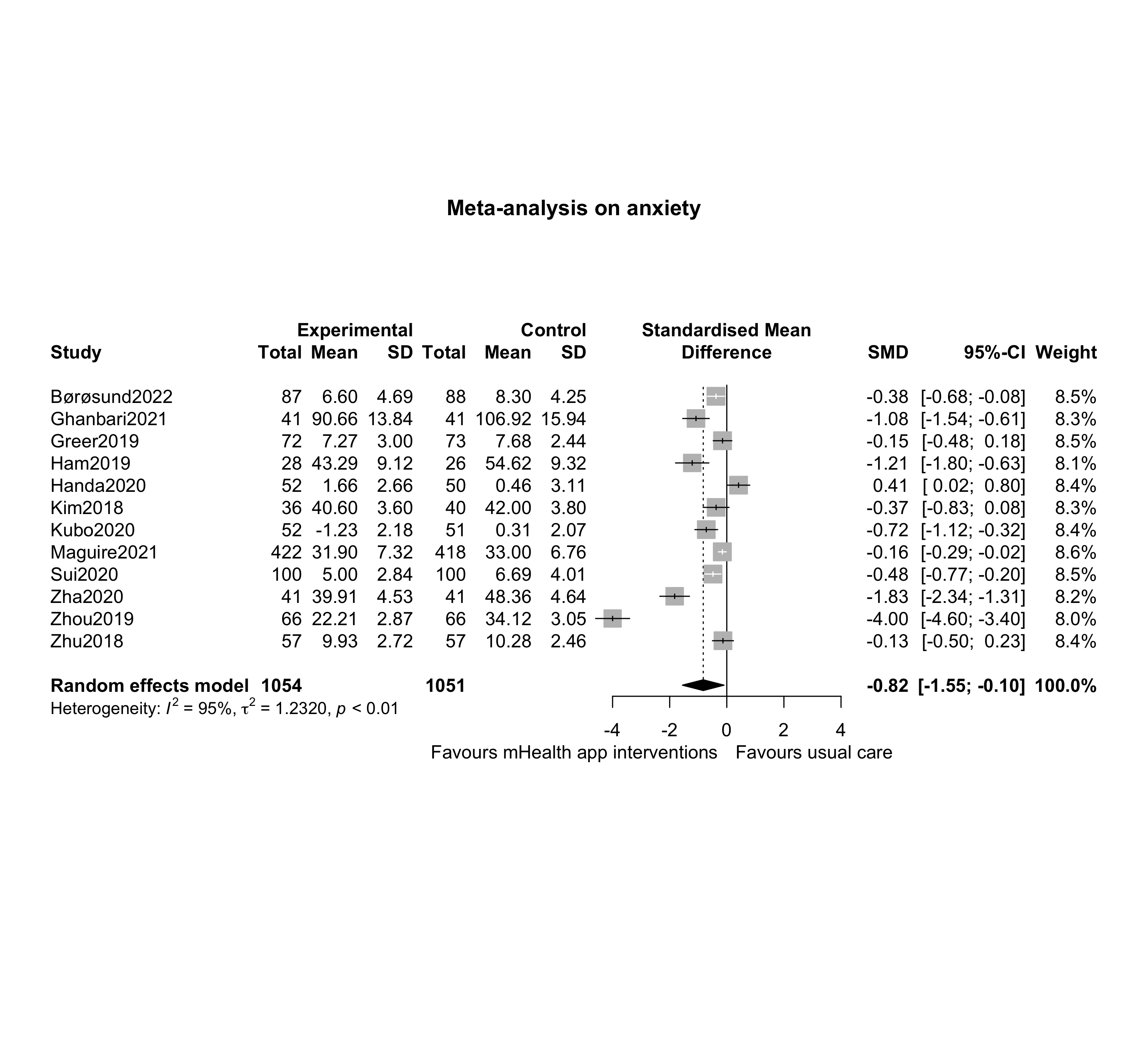

Supplement: S3 Fig — Meta-analysis on anxiety. (PNG) [file pone.0306772.s010.png]

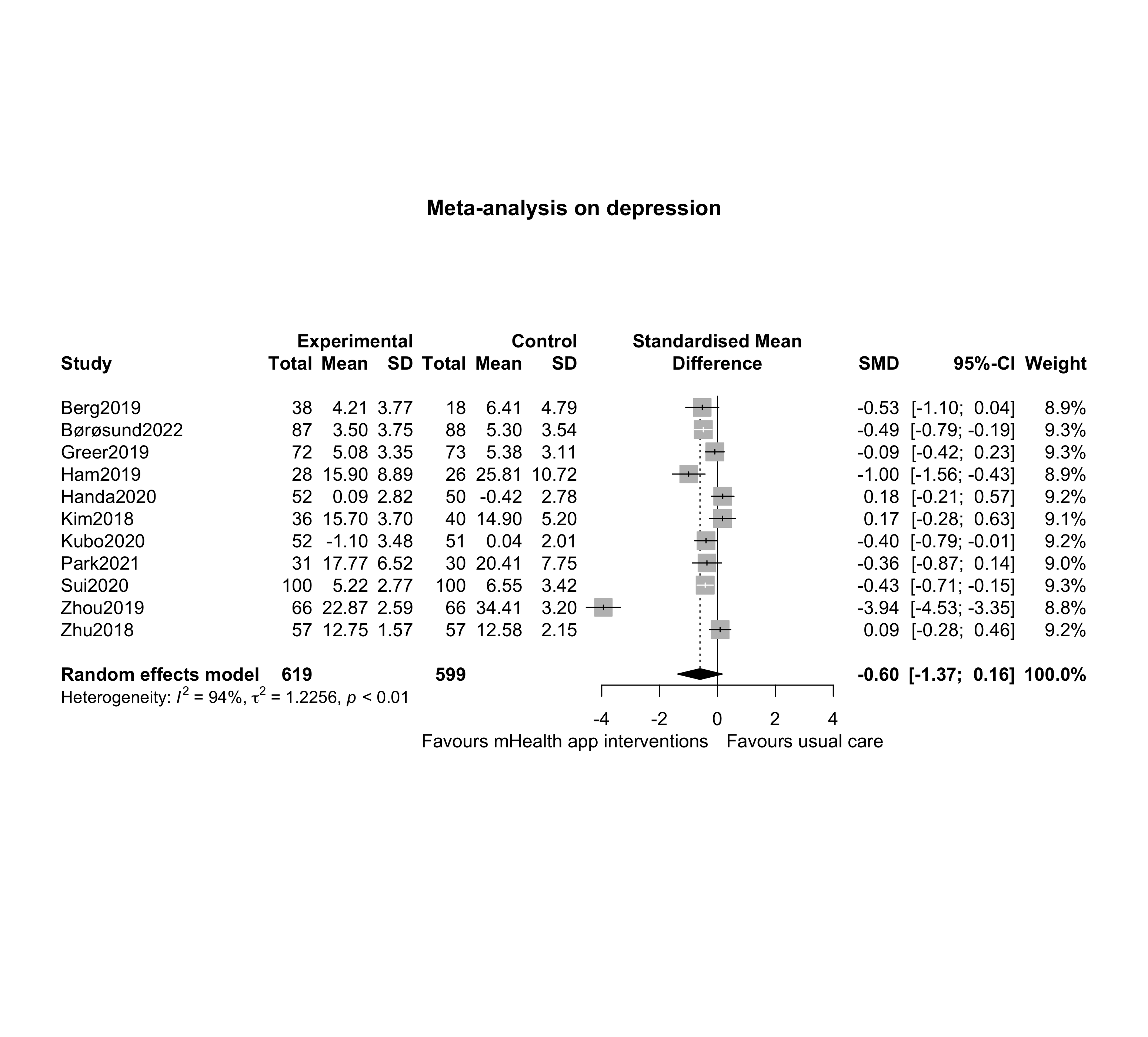

Supplement: S4 Fig — Meta-analysis on depression. (PNG) [file pone.0306772.s011.png]

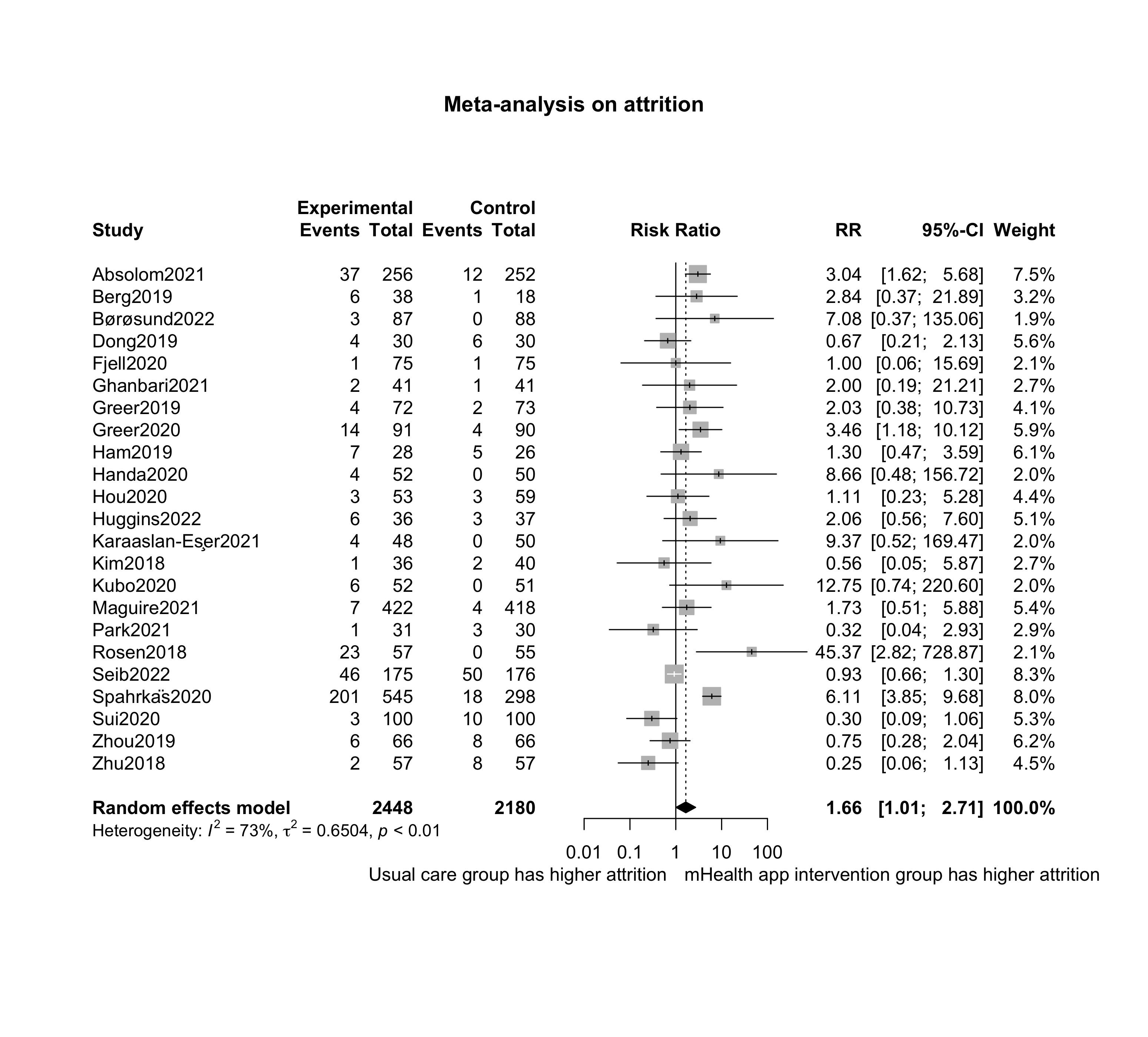

Supplement: S5 Fig — Meta-analysis on attrition. (PNG) [file pone.0306772.s012.png]
